# Effect of the PakCat program on nutrition status, dietary pattern and nutrition knowledge and skills of Pakistani women living in Catalonia evaluated by a mixed-method randomized control trial (RCT)

**DOI:** 10.1371/journal.pone.0316803

**Published:** 2025-01-14

**Authors:** Saba Mohamed-Bibi, Blanca Ribot-Serra, Cristina Vaqué-Crusellas

**Affiliations:** 1 Faculty of Geography and History, Department of Social Anthropology, University of Barcelona, Barcelona, Spain; 2 Faculty of Health Sciences and Welfare, Research Group M3O, Methodology, Methods, Models and Outcomes of Health and Social Sciences, University of Vic-Central University of Catalonia, Vic, Spain; University of Georgia, UNITED STATES OF AMERICA

## Abstract

**Background:**

Pakistani women are among the most affected groups by obesity and heart failure in Catalonia. Due to cultural and linguistic barriers, their participation in standard health promotion programs is limited. To address this issue, we implemented a culturally and linguistically appropriate food education program called the PakCat Program.

**Methods:**

The study employed a community-based participatory research (CBPR) design, utilizing a two-arm, cluster-assignment, non-blinded mixed-method randomized control trial (RCT) implemented in Badalona and Santa Coloma de Gramenet, two neighboring municipalities with a substantial Pakistani population. Participants were randomly assigned to the control group (n = 67) and the intervention group (n = 70). The intervention group received ten weekly culturally and linguistically appropriate food education sessions over ten weeks, while the control group attended three general sessions. The main outcome measures included nutritional status, nutrition knowledge and skills, dietary patterns, and satisfaction with the intervention, which were assessed pre-and post-intervention combining quantitative methods based on a survey and qualitative approaches consisting of conducting focus group discussions (FGDs).

**Results:**

The quantitative analysis conducted through a two-factor analysis of variance (ANOVA) for repeated measures indicated a significant improvement in the study variables across the entire sample (p < 0.001 within group), with the intervention group experiencing greater improvements in nutrition knowledge and skills and dietary pattern (p <0.001 for interaction and between groups). These findings were corroborated by thematically analyzed qualitative data confirming a more pronounced improvement in the study outcomes of the intervention group. Furthermore, both groups reported a high level of satisfaction with the intervention.

**Conclusions:**

The PakCat Program effectively improved the nutrition knowledge, skills, and dietary patterns of immigrant women of Pakistani origin residing in Catalonia. However, future research involving a larger sample size and combining the behavioral and clinical parameters is needed to enhance the generalizability of the results.

## Introduction

South Asians (SA) constitute one-quarter of the world’s population and account for over 50% of global cardiovascular deaths [1]. They are also affected by cardiovascular disease (CVD) at a younger age, presenting their first myocardial infarction approximately 10 years earlier than Caucasians [2]. Pakistan is the largest South Asian ethnic group in Catalonia (Spain), with a foreign resident population of 55,771 [3]. Women in this community are a minority (31%), mostly arriving in Catalonia due to the family reunification procedures predominantly sponsored by the male members of the family (husband or father) [4,5]. Upon their arrival, the men are already assimilated into the host society, however, the women encounter several cultural, linguistic, and bureaucratic barriers that convert them into one of the most invisible and vulnerable ethnic groups in Catalonia [5,6].

Apart from the language barriers derived from a lack of knowledge of the host country’s official languages (Spanish and Catalan), this invisibility is also attributed to the cultural division of gender roles that differ from the native society [7,8], as men take charge of the family’s financial matters while women remain relegated to the domestic and reproductive sphere, acquiring the role of family caregivers [5,7]. Furthermore, bureaucratic barriers, particularly the complex process of validating foreign qualifications, also impede the continuation of professional careers of some women who have higher education and work experience acquired from Pakistan [5]. Due to all these factors, social isolation becomes highly common among immigrant women of Pakistani origin [9,10], which, in addition to being related to a sedentary lifestyle, also contributes to the development of unhealthy eating habits [11–13] causing a deterioration in their health and well-being.

Studies show that the prevalence of CVD risk factors such as type 2 diabetes, hypertension, and obesity is higher among immigrant women of Pakistani origin in comparison with the male population of this community [14]. Furthermore, they are also among Catalonia’s most affected groups by obesity and heart failure compared to other South Asian groups and the indigenous populations of Catalonia [14]. Their social and health vulnerability evinces the need for gender-specific and culturally and linguistically sensitive health and nutrition interventions, which involve designing educational materials in their native language and recognizing their culinary customs and food-related traditions, to enhance their health outcomes and facilitate their integration into the host society [8,15]. Such initiatives have been implemented across various global contexts and have demonstrated efficacy in both the prevention and management of CVD risk factors along with the improvement of the eating habits of participants by promoting adherence to traditional dietary patterns based on plant origin, minimally processed, fresh, seasonal food [16–19]. However, these interventions are nonexistent in the Catalan context.

To address this gap, we designed the first culturally and linguistically appropriate food education program (PakCat Program) [19] considering the key success factors of the previous studies, such as co-creation, social networking, and community engagement [20,21]. The PakCat Program was based on the Transtheoretical Model [22] and aimed to improve the eating habits of Pakistani women living in Catalonia, while also empowering them to become ambassadors of healthy eating habits in their community. This paper illustrates the evaluation of the effectiveness of the PakCat Program based on the participants’ nutritional status, nutrition knowledge and skills, dietary pattern, and satisfaction with the intervention.

## Materials and methods

### Trial design

Community-based participatory research (CBPR) in which a two-arm cluster-assignment, non-blinded mixed-method randomized control trial (RCT) with a 1:1 allocation ratio was implemented.

### Participants, eligibility criteria, and settings

The study was conducted in the province of Barcelona, home to 87% of the foreign population of Pakistani origin residing in Catalonia. Explicitly, in Badalona and Santa Coloma de Gramenet, two neighboring municipalities and, respectively, the second and fourth most populous in Catalonia by the Pakistani population. The primary interaction points of immigrant women of Pakistani origin residing in these municipalities were the *Fundació Ateneu Santa Roc* (Badalona) and *Casa Àsia* (Santa Coloma de Gramenet), two institutions that work to facilitate the integration of migrant people into the host country and its environment. Each institution annually attends between 50 and 60 Pakistani adult women.

We invited both institutions to participate in the study, and they agreed to collaborate and facilitated the recruitment of participants (which began on June 30th, 2021, and ended on December 20th, 2021), allowing us to invite all their Pakistani students and their close acquaintances to participate. We conducted several introductory sessions for both the personnel and the interested participants from these institutions to explain in depth all the characteristics of the project.

Any adult (>18 years) immigrant woman of Pakistani origin with residence in Badalona and Santa Coloma de Gramenet volunteering to participate in the study was included in the study. Women with a diagnosis of cognitive impairment or any physical illness that could make participation in the study difficult and those who did not agree with the ethical conditions of the study were excluded.

**Fig 1:**
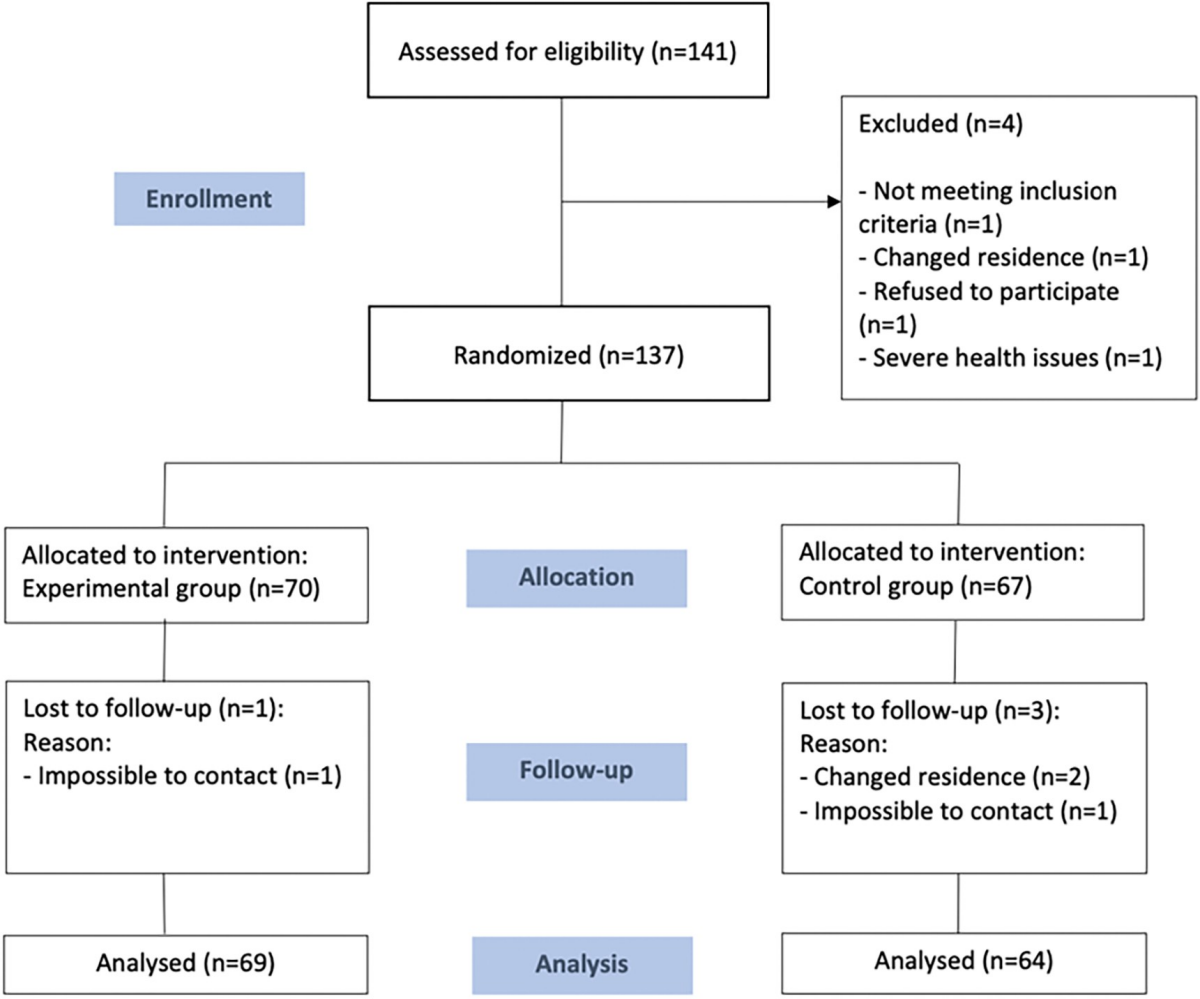
Consort flowchart.

A total of 141 women (71 from *Casa Àsia* and 70 from *Fundació Ateneu Sant Roc*) were recruited using a non-probabilistic consecutive sampling method (Fig 1). One institution was assigned as the intervention group and the other as the control group, to avoid possible contamination of information between participants.

The trial is registered as ISRCTN15670247 (https://www.isrctn.com/ISRCTN15670247). The reason for registering it retrospectively was a misconception, since we believed that community RCTs did not require registration. This CBPR, RCT, was also approved by the Bioethics Committee of the University of Barcelona (CBUB). All participants provided written informed consent. The research protocol has been previously published [19]. We adhered to the CONSORT (Consolidated Standards of Reporting Trials) 2010 guidelines for reporting this RCT [23].

### Intervention

The 10-week intervention was developed in small subgroups of women (12–15 women). The intervention group participated in 10 educational sessions based on the Transtheoretical model (Table 1) for 10 weeks, while the control group attended three general sessions on healthy eating. Each weekly session had a duration of 90 minutes. A multilingual nutritionist of Pakistani origin carried out the sessions in Urdu and Punjabi at different community spaces. The objectives, methodology, and final activities of each session have been previously published [19].

**Table 1:** Food education sessions based on the Transtheoretical model.

Stage of change	Objective	Process of change	Change strategy	Session
**Pre-contemplation**	Raise awareness of the problem by stimulating the possibility of change	*Consciousness raising*	Present and discuss food and lifestyle changes due to migration.	1. Why us? The most common health problems of the Pakistani population 2. Food myths and beliefs: What does science say?
		*Dramatic relief*	Present data on the prevalence of CVD in immigrants of SA origin.	
		*Self-reevaluation*	Focus group on the strengths and weaknesses of the dietary pattern.	
			Debate on myths and beliefs related to food and health.	
		*Environmental reevaluation*	Highlight the impact of their eating habits on future generations	
**Contemplation**	Decant the scale towards change	*Consciousness raising*	Present the benefits of following healthy eating.	3. What is a healthy diet? 4. The value of our traditional diet
			Introduce the disadvantages of following an unhealthy diet.	
		*Self-reevaluation*	Self-assessment of current eating habits by compiling a list of healthy and unhealthy habits.	
**Preparation**	Reinforce knowledge to facilitate the change	*Counterconditioning*	Conduct group sessions on food or behaviors that need to be enhanced, reduced, or changed regarding the type or quality, to follow a healthy diet.	5. Small changes to eat better (MORE) 6. Small changes to eat better (CHANGE) 7. Small changes to eat better (LESS)
		*Self-liberation*	Collect suggestions to acquire the recommended changes.	
**Action**	Effectuate the change by promoting self-efficiency	*Stimulus or environmental control*	Workshops on dietary planning and food purchasing; elaboration of healthy dishes with traditional foods; interpretation of nutrition labeling, preparation of healthy breakfasts and snacks.	8. Let’s plan our weekly food purchase! 9. How to plan a balanced menu?
**Maintenance**	Maintain the change	*Helping relationships*	Photovoice exhibition of healthy plates [18]	10. Photovoice
		*Social liberation*	Acquisition of the role of promoting agent of healthy eating habits for the rest of the community.	

The intervention material was also elaborated in Urdu, Catalan, Spanish, and English languages. The elaboration procedures of the intervention materials, along with the related themes, have been detailed and published in prior works [24].

The intervention started with 137 participants and ended with 133 participants (Fig 1). A total of 69 participants from the intervention group attended six or more sessions, while 64 participants from the control group attended a minimum of two sessions.

### Study outcomes

The main outcomes of the study were nutritional status, nutrition knowledge and skills, dietary patterns, and satisfaction with the intervention. These outcomes were measured by combining both qualitative and quantitative methods (Table 2).

**Table 2:** Data collection methods through qualitative and quantitative approaches.

Outcomes	Quantitative method	Qualitative method
**Sociodemographic data**	The sociodemographic data identified through the survey were age, place of birth, marital status, academic studies, employment, languages, religion, the reason for migration, years of residence in Catalonia, and household members and their professions.	The sociodemographic data were only collected through quantitative method.
**Nutritional status**	The nutritional status was defined by anthropometric measurements (weight, height, and waist). Waist circumference was assessed at the midpoint between the lowest rib and the iliac crest with the precision recorded to the nearest 0.1 cm while the person was standing using non-stretchable tape. Body weight was measured in kilograms in light clothing, without shoes and pocket emptied using a scale (seca 896) and height was measured to the nearest 0.5 cm with a portable stadiometer (seca 213).	During the FGDs, participants discussed about changes in their clothing size and compliments about weight reduction from their close acquaintances.
**Nutrition knowledge**	The survey included 15 true/false questions about nutritional knowledge such as the ability to distinguish different types of fats, familiarity with different sources of nutrients, knowledge about portion and frequency of consumption of different food groups, number of meals daily meals, ability to read and interpret food labels, and food-related myths and beliefs [18,25,26]. These questions were recodified as follow: correct answer = 1 point and incorrect answer = 0 points. The answers were totaled, and a higher score was indication of a better nutrition knowledge.	During the FGDs participants were asked about their nutrition knowledge, including their understanding of the different food groups and their recommended frequency of consumption along with their impact on our health and well-being. The FGDs also provided information related to the food beliefs of participants including their opinion about herbal products and the traditional myths related to food such as classifying the food in cold and hot categories.
**Nutrition skills**	The survey also contained 13 multiple-choice questions about nutritional skills such as menu planning, preparing a grocery list, differentiating various food groups, having knowledge about seasonal food, reading and interpreting food labels, preparing food with different culinary techniques, reusing food leftovers, and preparing a healthy dish. These questions could be answered as follows: no difficulty (1 point), little difficulty (2 points), some difficulty (3 points), quite a bit of difficulty (4 points), and a lot of difficulties (5 points). The answers were totaled, with a lower score indicating greater dietary skills [26].	The FGDs also included questions about the nutrition skills of participants such as menu planning, culinary techniques, ability to vary the diet, use of food alternatives, reading and interpreting food labels and elaboration of the healthy plate.
**Dietary pattern**	The survey also included an 18-item Food Frequency Questionnaire (FFQ) adapted from the Table of Indicative Frequencies of the Spanish Society of Community Nutrition (SENC) [27]. The consumption of 18 food groups was determined through serves/day/week/month with the help of a practical guide on portion size for the Pakistani population. Therefore, when the SENC’s recommended frequency of consumption for a food group was followed, 1 point was assigned, whereas non-compliance resulted in 0 points. For example: having 2 servings of vegetables daily—Compliance = 1 point, non-compliance = 0 point. The answers were totaled, and a higher score indicated a better dietary pattern.	During the FGDs, participants were asked about the timing, number and structure of meals. We also asked about the frequency of consumption of different food groups.
**Satisfaction with the intervention**	The satisfaction with the intervention was only assessed through qualitative method.	Participants were asked to explain their experience with the intervention.

### Participant timeline

The total duration of participants’ involvement in this research was approximately 7 months for the intervention group (2 months for baseline data collection, 3 months for the intervention implementation, and 2 months for the post-intervention assessment) and 5 months for the control group (2 months for baseline data collection, 1 month for the intervention implementation, and 2 months for the post-intervention assessment).

### Sample size

Due to the scarcity of data regarding changes in nutrition knowledge, skills, and dietary patterns among Pakistani women participating in nutrition education programs, traditional sample size calculations based on specific statistical tests, expected effect sizes, and primary response variables were not feasible. Additionally, significant barriers to access and participation of this population in research projects, a non-probability convenience sampling method was employed. This approach involved including all eligible women who agreed to participate during the study period, resulting in a final sample of 141 participants.

We acknowledge the limitation of not conducting a formal sample size calculation and assess its relevance by comparing it with similar studies conducted in Pakistani immigrant communities, which typically involve sample sizes ranging from 150 to 200 participants [17,28]. The decision to adopt this approach is aligned with the exploratory nature of the study and underscores the need to establish an initial database for future research within this population.

### Randomization

To ensure randomization in 1:1 allocation, an external researcher who was not participating in the study wrote the names of both entities on separate pieces of paper and placed them into sealed, opaque envelopes. To maintain allocation concealment, the envelopes were stored by an administrative staff member not involved in the study. When both clusters were ready for the assignment, the external researcher randomly selected and opened one envelope in the presence of the research team. The entity named inside the first opened envelope was assigned to the intervention group, while the other became the control group. This procedure ensured an unbiased allocation, enabling random assignment between the two groups.

### Blinding

This was a non-binding study. Both the investigator and participants had knowledge of their assignment to the control and the intervention groups.

### Data collection

The data was collected through a mixed-method approach that involved combining both quantitative and qualitative methods. The baseline assessment, conducted before implementing the intervention, consisted of answering a survey in Urdu, that included 5 main sections: sociodemographic data, anthropometric data, a questionnaire about nutrition knowledge, a questionnaire about nutrition skills, and a Food Frequency Questionnaire (FFQ), (Table 2).

The qualitative method consisted of conducting focus group discussions (FGDs) to: explore the interest of participating women in health and nutritional aspects, understand their current and traditional dietary patterns, identify their nutritional beliefs, knowledge, and skills, analyze their role in making dietary decisions, discover the factors that could facilitate or hinder the adoption of a healthy lifestyle; and comprehend their expectations and demands towards the program. In this way, the FGDs allowed the co-creation of an intervention adapted to participants’ needs and circumstances.

We conducted six FGDs, three in both municipalities, each with six participants (n = 36). The discussions lasted between 70–90 minutes. The design and implementation of focus groups followed the procedures set out by Krueger [29]. A multilingual nutritionist of Pakistani origin conducted the FGDs in Urdu language using a semi-structured guide developed after reviewing the literature on diet-related knowledge, attitudes, and behaviors of immigrant women of Pakistani origin [16–18]. Comprehensive information regarding the execution of pre-intervention FGD has been previously published [30].

Post-intervention data collection was carried out immediately after the completion of the nutritional education sessions, and it consisted of gathering the same variables that were collected during the baseline data collection, combining both qualitative and quantitative methods. Thus, the survey was repeated, and we reconducted six FGDs (3 with the intervention group and 3 with the control group) to comprehend in depth the changes in the study variables and determine whether the intervention fulfilled participants’ expectations.

### Data analysis

The quantitative data were analyzed with SPSS version 27.0. To assess the baseline characteristics a descriptive analysis was performed (Table 3). To compare the profiles of the control and intervention groups, we assessed the normality of continuous variables and employed a T-test for normally distributed variables and a Mann-Whitney U test for non-normally distributed variables (Table 3). For categorical variables, Chi-square tests and Fisher’s exact tests were used when appropriate (Table 3).

**Table 3:** Basic characteristics of participants.

Basic characteristics of participants	Intervention group	Control group	Bilateral significance	Basic characteristics of participants	Intervention group	Control group	Bilateral significance
**Age (years)** Mean (SD) Range	37.4 (10.9) 19–63	37.4 (10.7) 18–60	0.99^a^	**Years of residence in Catalonia** Median [P_25_-P_75_] Range	7.0 [2.8–10.0] 0.2–19	5.0 [1.0–10.0] 0.3–23	0.40 ^b^
**Marital status** Single Married Divorced Widowed	13(18.6%) 52 (74.3%) 3 (4.3%) 2 (2.9%)	11 (16.4%) 54 (80.6%) 1 (1.5%) 1 (1.5%)	0.71^c^	**Reason for migration** Family reunification Others	67 (95.7%) 3 (4.3%)	67 (100%) 0 (0%)	0.25 ^d^
**Education** No qualifications Early childhood education Primary education Secondary education Higher education (college/university)	0 (0%) 6 (8.6%) 1 (1.4%) 23 (32.9%) 40 (57.1%)	2 (3%) 4 (6%) 7 (10.4%) 17 (25.4%) 37 (55.3%)	0.94 ^c^	**Languages** Urdu and Punjabi Urdu, Punjabi, and English Urdu, Punjabi, English & Spanish Urdu, Punjabi, English, Spanish & Catalan	23 (32.9%) 19 (27.1%) 26 (37.1%) 2 (2.9%)	22 (32.8%) 20 (29.9%) 17 (25.4%) 8 (11.9%)	0.69 ^c^
**Profession** Teacher Health professional Housewife Supermarket cashier Others	9 (12.9%) 2 (2.9%) 47 (67.1%) 6 (8.6%) 6 (8.6%)	12 (17.9%) 4 (6%) 43 (62.7%) 3 (4.5%) 6 (9%)	0.35 ^c^	**Household members** 1–3 persons 4–6 persons > 6 persons	10 (14.3%) 44 (62.9%) 16 (22.9%)	12 (17.9%) 37 (55.2%) 18 (26.9%)	0.97 ^c^
**Occupation** Occupied Unoccupied	7 (10%) 63 (90%)	10 (14.9%) 57 (85.1%)	0.27 ^d^	**Working members of the household** Median [P_25_-P_75_] Range	1.0 [1.0–2.0] 0–4	1.0 [1.0–2.0] 0–5	0.86 ^b^

SD: Standard deviation; ^a^ T-test; ^b^ Mann-Whitney U test; ^c^ Chi-square test, ^d^ Fisher test.

The distributions of pre-post differences for each variable were evaluated and found to approximate normality, validating the use of parametric tests for the analysis. To compare the study variables pre- and post-intervention based on the study group, a two-factor analysis of variance (ANOVA) for repeated measures was employed, considering time as an intra-subject factor and group as an intersubject factor (with two levels: control versus intervention), along with their interaction.

The post-intervention qualitative data were analyzed thematically with Atlas.ti version 22. We adhered to Krueger’s indications for the transcription and translation of the focus groups. The audio recordings were first transcribed verbatim into Urdu and then translated into Catalan. The accuracy of translations was assessed and approved by another multilingual South Asian health professional. The transcripts were thematically analyzed using the six phases of Braun and Clarke’s guidelines for the analysis [31]. After getting familiarized with the data, our multilingual research team individually performed open coding and met to discuss the initial interpretations. Hereafter, initial thematic maps were created to identify potential themes. Several meetings were organized to discuss and refine the initial themes. At first, themes were created inductively, and then considering the variables of the study related to the efficacy of the intervention, a deductive approach was followed. These outcomes were defined as themes and similar codes were organized into subthemes.

## Results

The analysis of basic characteristics indicated no statistically significant differences, confirming the homogeneity of the groups (Table 3).

The following are the outcomes related to the efficacy of the intervention analyzed through quantitative and qualitative methods:

### Nutritional status

#### Quantitative method

**Fig 2:**
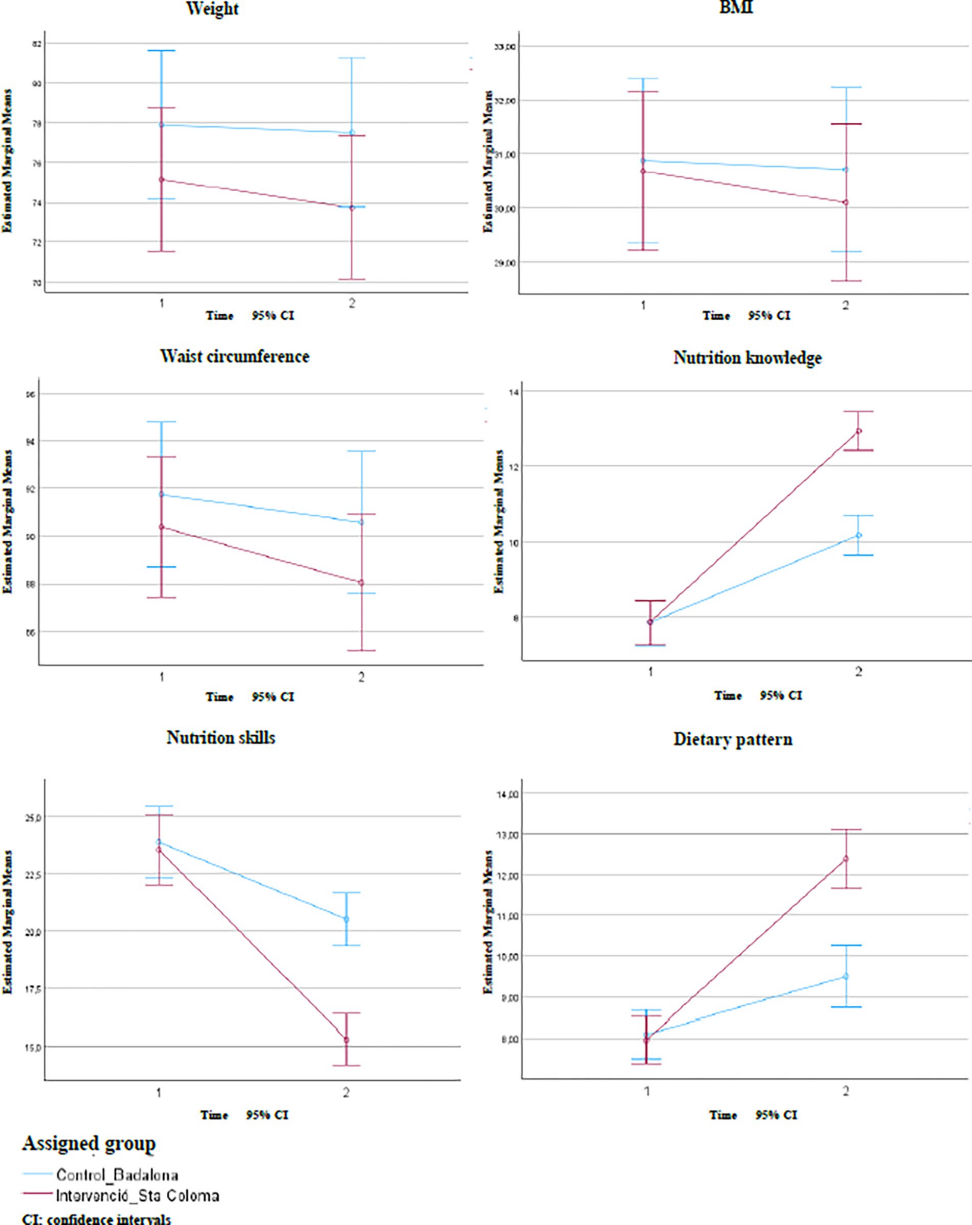
Time*Group interaction on study variables–changes in control and intervention group over time.

After the intervention, statistically significant reductions in weight, BMI, and waist circumference were observed over time in both the control and the intervention group (p<0.001) (Table 4). However, the reductions were more pronounced in the intervention group with a mean weight loss of -1.41 kg compared to -0.37 kg of the control group, with corresponding BMI reductions of -0.58 and -0.16, respectively (Table 4). The weight and BMI interaction effects were also significant (p <0.05), suggesting a greater reduction in the intervention group. Although the intervention group also showed a larger decrease in waist circumference than the control group, the interaction effect was not significant, indicating similar changes in both groups (Table 4; Fig 2). The non-significant between-groups effect (p-value >0.05) indicates that the overall reduction in these three parameters did not significantly differ between groups (Table 4).

**Table 4:** Comparison between nutrition status, nutrition knowledge, nutrition skills and dietary pattern.

	Pre-intervention	Post-intervention	P-value Within groups (time)	P-value interaction (time*group)	P-value Between groups
Weight (Kg), Mean (SD) Control group Intervention group	77.89 (14.34) 75.15 (15.54)	77.52 (14.30) 73.74 (15.57)	<0.001	0,034	0,214
BMI (kg/m2), Mean (SD) Control group Intervention group	30.87 (5.90) 30.68 (6.33)	30.71 (5.79) 30.10 (6.33)	<0.001	<0.001	0,704
Waist circumference (cm), Mean (SD) Control group Intervention group	91.74 (12.01) 90.37 (12.47)	90.57 (12.23) 88.03 (11.69)	<0.001	0.103	0.350
Nutrition Knowledge (points), Mean (SD) Control group Intervention group	7.86 (2.55) 7.87 (2.62)	10.17 (2.68) 12.94 (1.53)	<0.001	<0.001	<0.001
Nutrition skills (points), Mean (SD) Control group Intervention group	23.89 (6.31) 23.55 (6.41)	20.51 (5.29) 15.27 (4.07)	<0.001	<0.001	<0.001
Dietary pattern (points), Mean (SD) Control group Intervention group	8.09 (2.53) 7.96 (2.29)	9.50 (2.94) 12.39 (3.14)	<0.001	<0.001	<0.001

#### Qualitative method

We identified two subthemes related to the nutritional status:

1. Reduction in clothing size: In the post-intervention FGDs, most women from the intervention group reported a decrease in their clothing size, while participants from the control group did not mention any changes (Table 5).
2. Compliments from the social circle: Many participants from the intervention group reported receiving compliments from their social circle on their weight loss. Conversely, the control group barely discussed the feedback from their social circle regarding the decrease in their weight.

**Table 5:** Emerging themes and example quotes from the control and the intervention group.

Theme	Subtheme	Example quotes from the intervention group	Example quotes from the control group
Nutritional status	Reduction in clothing size	S37: I also feel lighter, I feel like I’ve lost weight. Clothes that used to be too tight for me now fit perfectly. Now I feel like I’m getting better.	N37: Believe me, it was December and even my XL jacket didn’t fit me. I have decided that I won’t buy a new one, I will lose weight and wear the same one.
	Compliments from the social circle	Z32: My sister-in-law was telling me the other day that she has noticed that I’ve lost some weight and asked me how I did it.	S35: Now my daughters say: “Mom! Now when you walk with us you look so tall and slim!”
Nutrition knowledge	Knowledge about food groups	A35: In addition, we can take good fats but in moderation. For example, avocado, we can add it to our salads, and nuts also have good fat. We can also use even 6 tablespoons of olive oil per day.	B40: That’s why we should know the groups of things in which we can find protein, the group of vitamins…Mineral, I think, water and juice have minerals? But we need to learn about it.
	Knowledge about the recommended portion and frequency of consumption	Z30: Before we used to fill the plate with rice only but now, we prepare a healthy plate. H45: Half of a healthy plate should be vegetables. Moderator: But in one day. . . H45: Twice! Z30: Yes, one raw, one cooked (vegetable)! Moderator: And the fruit? Z30, M33 & H45: Three times! N50: And white meat, chicken, fish. . . 2–3 times chicken and 2–3 times fish, in a week.	N30: But when it comes to making the plate, it’s still not clear to me. The amount, I mean, what portion should be of all this? I know that of rice and chapati, I have to choose one! Or rice or chapati, that’s clear to me. If there is potato, then there is no need for chapati. But there are things I still don’t know, if I take eggs what should be the amount, and then can I eat the meat curry too? I don’t know that yet.
	Demystification of food-related myths and beliefs	A35: Exactly, in the past, I had two miscarriages and I strictly used to follow hot and cold foods (theory). I used to avoid hot foods (during pregnancy) because everyone suggested not to take them, but now I eat fish, eggs. Fruit, milk, everything! Thank God, I am very well, I am healthy! Also, I believe I got pregnant thanks to the healthy diet and all naturally.	M48: Yes! That’s true. For example, F46 says that it is difficult for her to digest watermelon, this is because she finds it cold. If you accompany it with dates (which are considered a hot food), you will be able to combat the cold effect of the watermelon and produce a balance. So, you will digest the watermelon.
Nutrition skills	Menu planning and preparation	N50: I notice that if today we have prepared the meat, then tomorrow we will prepare the vegetables, then the lentils. . .	N42: We should keep in mind that repeating same food group is not good. We have to think that if we have already eaten a group for breakfast, then we don’t repeat it for lunch.
	Ability to vary diet	H50: For example, our typical fruit chaat (fruit salad). Now we add yoghurt to it. Before, I used to add the apricot sauce with sugar and milk cream. But now I don’t use creams or anything. Just yoghurt with a little black pepper, salt, sesame (seeds), nuts. . . I have also prepared a powder of nuts and seeds.	B40: I only know how to fry fish; I tried making it in the oven, but it didn’t work. Now I’m learning how to roast it. N40: We actually bought a machine! Air fryer! We prepare chicken, kebabs. . . Nothing fried. Everyone happy!
	Reading and understanding of food labels	A33: Now I check the amount of salt and sugar. M28: We also read the ingredients. The first ingredients are found in the largest amount in the product.	S37: Yes, to the point that now when I go shopping, what you told us about salt and sugar, I focus on that. Products with more salt, even those with 0.23–0.24 grams, I do not buy.
Dietary pattern	Improvement in timing, frequency, and structure of meals	H50: Now we have 5 meals. We have breakfast in a limited amount and then at 10am we eat a piece of fruit and, then, lunch also in a limited amount. I, personally, now, digest well. I feel by this way digestion improves a lot. Then at 5 or 6 p.m., we have a place to eat something. Then around 7 or 8 p.m., we can have dinner. Before, we used to have lunch around 4 or 5pm and fill all our stomach. Afterwards we did not want to have dinner and could not digest all that we had eaten.	S35: We used to eat a lot of food in only one meal. So much! To the point that after our meals, it was difficult for us to even move. S40: Yes, when I used to do this, I felt like I was carrying a weight inside. Now, I feel very light N50: I also feel very light now, I have a routine, a schedule! B40: She (her sister) used to eat rice for breakfast, lunch, and dinner! She used to cook it in a very big pot! She said I can’t cook 3 times a day, I will cook once and that’s it! N37: Now I have stopped doing this.
	Increase in the consumption of wholegrain products	S37: We also use 100% wholemeal flour. M28: I also use 100% wholemeal flour and it is a very coarse flour, and with this flour you cannot eat a very large chapati. A small chapati of wholemeal flour is enough, it is much more filling. My children, husband, really liked it.	M48: People who have always used refined flour find this change very difficult. We first went to number 90 (semi-wholegrain flour) and now we will gradually move to 100 (wholegrain flour).”
	Increase in the consumption of fruit, vegetables, and nuts	Z32: (Before) For lunch chapati, for dinner chapati, only chapati with curries! We didn’t eat fruit or vegetables. . . And before I only ate an apple, every day! Now I try to vary and eat 3 pieces of fruit every day.	N30: For example, I’m too lazy to go shopping. Like the salad, if it’s finished, I’ll have a hard time going shopping. That’s why I don’t follow (a healthy diet).
	Reduction in the consumption of red meat	S35: Before we used to prepare kebabs with minced meat. . . H50: Yes, we ate a lot of Red Meat. Now we have reduced it. S35: Now we make kebabs with chickpeas or chicken.	M48: We eat seasonal vegetables. We can add any type of meat. Whatever we like, be it chicken, mutton, beef. . .doesn’t matter.
	Reduction in processed and ultra-processed food	F43: Since I started attending the sessions, I stopped eating cookies. I no longer accompany them with tea. S37: Obviously! They (children) copy us. Look, we’ve stopped buying Coke! If we leave, they will also leave. M28: I don’t buy Coke either. H50: I don’t even buy juices.	S35: I also used a lot of breakfast cereals, the ones with a bit of fruit, but now that I have seen that they have a lot of sugar, I no longer buy them. R26: I have stopped eating fast food and drinks.
	Lower use of butter and refined oil	Z30: We changed the oil too! Now we use olive oil. A33: And instead of ghee, I add olive oil, instead of sugar, dates! (to a traditional dish) I63: I also use olive oil for cooking.	N42: Olive oil is hot. S35: Yes! N42: When I use olive oil, my skin starts itching. . . S35: But when I add the olive oil to the salad, it doesn’t feel hot, but for cooking it’s not good, it feels very hot.
**Satisfaction with the intervention**	Meeting expectations and enhancing confidence	S37: Yes, before when we went shopping, he (my husband) would say: "Let’s buy this, that. This is good, this is not!” Now I explain all this to him, in details, and he listens to me! F43: We even explain it to our relatives in Pakistan.	S37: I feel very knowledgeable! I mean I feel empowered. It has benefited me a lot. S28: We feel very good when we share all this knowledge with our cousins, sisters-in-law. . .

### Nutrition knowledge

#### Quantitative method

The nutrition knowledge of both the control and the intervention group was significantly improved, as suggested by an increase in the means of both groups along with a significant within-group effect (p <0.001) (Table 4). However, the increase in nutrition knowledge was significantly higher in the intervention group (p<0.001) (Table 4; Fig 2).

#### Qualitative method

During the FGDs, we observed that both the control and intervention groups gained knowledge about the basics of healthy eating. However, we observed some differences in subthemes:

1. Knowledge about food groups: The women from the control group were not familiar with the concept of nutrients and their sources, however, participants from the intervention group, demonstrated a more comprehensive understanding of this information and were also able to differentiate between various types of carbohydrates and fats (Table 5).
2. Knowledge about the recommended portion and frequency of consumption: In contrast to the women in the control group, participants from the intervention group were aware of the recommended portions and frequency of consumption of different food groups. They particularly highlighted the reduction in the portion of carbohydrates, which are traditionally consumed in excess.
3. Demystification of food-related myths and beliefs: Participants from the intervention group were able to demystify many food-related myths and beliefs including the classification of food according to its thermogenic effect (hot/cold), the assumption that homemade juices are healthy, the belief that cooked or frozen vegetables lose all vitamins and minerals, the myth about following a hyperlipidic diet during pregnancy, the harmful effects of restrictive diets on our health, and the belief that herbal infusions help to reduce weight. However, the control group held on to these beliefs (Table 5).

### Nutrition skills

#### Quantitative method

Participants in both the control and intervention groups significantly improved their nutrition skills as the means of both groups decreased, and the within-groups time effect also resulted in significant (p <0.001) indicating a substantial improvement in nutrition skills of the entire sample (Table 4). However, the intervention group exhibited a greater enhancement in nutrition skills, as evidenced by a lower mean score, a significant time-group interaction effect (p <0.001), and a significant between-group effect (Table 4; Fig 2).

#### Qualitative method

During the FGDs, participants from both groups reported an improvement in their nutrition skills. However, the changes were more noticeable in the intervention group.

1. Menu planning and preparation: Participants from the intervention group explained that they have learned to plan daily/weekly healthy menus. They have also acquired skills in the preparation and complementation of healthy dishes following their traditional dietary pattern as well as preparing different healthy breakfasts and snacks. The control group stated that they still lack these skills (Table 5).
2. Ability to vary diet: Women from the intervention group mentioned that they have developed abilities to diversify their diet, utilize healthier food alternatives (replace red meat with legumes, sugar, and sweeteners with dates, bananas, etc.), and prepare traditional dishes in a healthy format. They have also learned and incorporated some new cooking techniques (especially oven and steam) (Table 5).
  Although the control group reported some new culinary skills, they stated difficulties in healthily preparing traditional dishes and diversifying their diet.
3. Reading and understanding of food labels: Both groups reported improvements in reading and interpreting the food labels and confirmed reviewing nutritional labels before purchasing food products (Table 5).

### Dietary pattern

#### Quantitative method

Participants in both groups demonstrated significant improvements in their dietary patterns, as indicated by increases in mean scores and a significant within-groups time effect (p <0.001) (Table 4).

However, the changes were more pronounced in the intervention group demonstrated by a greater mean score, a significant time-group interaction effect (p-value <0.05), and a significant between-group effect (Table 4; Fig 2).

#### Qualitative method

The intervention group mentioned more prominent changes in their dietary pattern as compared to the control group (Table 5).

1. Improvement in timing, frequency, and structure of meals: Participants from both the control and the intervention groups reported establishing regular mealtimes and eating 4–5 meals a day. Moreover, the participants from the intervention group additionally reported improved meal structure consisting of including protein in every meal and reducing the amount of carbohydrates, aligning with the correct portion size. They affirmed that implementing a healthy plate structure for lunch and dinner is effective in following the recommended portion of different food groups. However, the control group recognized the persistent challenges in modifying their meal structure.
2. Increase in the consumption of whole-grain products: The intervention group explained that they had successfully replaced refined flour with whole-grain flour, whereas the control group reported difficulties in implementing this change (Table 5).
3. Increase in the consumption of fruit, vegetables, and nuts: Participants from the intervention group mentioned that they have increased their consumption of fruit, vegetables, and nuts, however, most participants from the control group stated that they have not succeeded in achieving the aforementioned changes (Table 5).
4. Reduction in the consumption of red meat: The intervention group reported a reduction in red meat intake, replacing it with legumes, lean meat, and fish. They also stated the inclusion of protein in every meal, especially for lunch and dinner. These modifications were not mentioned by the control group (Table 5).
5. Reduction in processed and ultra-processed: Participants from both the control and intervention groups stated a reduced consumption of carbonated drinks, juices, fast food, pre-cooked foods, salty snacks, and industrial or homemade bakery products (Table 5).
6. Lower use of butter and refined oil

Women from the intervention group reported lower consumption of butter and refined oil, prioritizing the use of olive oil, however, most participants from the control group were not using olive oil as their main dietary fat (Table 5).

### Satisfaction with the intervention

#### Qualitative method

Participants from both the intervention and control groups expressed satisfaction with the intervention.

1. Meeting expectations and enhancing confidence: Both the control and intervention groups stated that the intervention met their expectations, enabling them to improve their eating habits, nutritional knowledge, and skills, along with their nutritional status. Participants from both groups expressed feeling empowered to pass on the gained nutrition knowledge to their families. Furthermore, they indicated that the intervention has allowed them to strengthen their social connections and community participation.

## Discussion

The results of the present study indicate that a culturally and linguistically appropriate nutrition education program can improve the nutrition status, eating habits, and nutrition knowledge and skills of Pakistani women living in Catalonia. In this RCT, 137 Pakistani women from two different municipalities were randomly assigned to the control and intervention groups. As both groups were placed in different locations, data contamination between them was minimized. The combination of quantitative and qualitative approaches in data collection and analysis enabled a more comprehensive exploration of the study variables.

After the intervention, the weight changes observed in the intervention group adhered to the 0.5–2.5 kg weight loss range (Table 4), which is associated with favorable effects on metabolic variables, especially when combined with decreased waist circumference and increased physical activity [32,33]. However, as our study lacked information on clinical parameters and physical activity levels, the clinical relevance of these improvements remains uncertain.

Furthermore, while the intervention group exhibited a more pronounced decrease in weight and BMI compared to the control group, these differences were insufficient to provide a significant between-group effect (p >0.05). These results align with the InnvaDiab-DE-PLAN study[17]; an RCT conducted with 198 Pakistani women residing in Norway, which apart from combining nutrition education with physical activity practice, also included an assessment of clinical parameters related to the risk of type 2 diabetes. This study also found no statistically significant (p = 0.183) difference between groups in weight over 7 months, even though the intervention group achieved significant weight loss (p = 0.013).

The same was observed in culturally and linguistically adapted lifestyle interventions implemented in immigrants of South Asian origin. In the Prevention of Diabetes and Obesity in South Asians (PODOSA) study, a culturally and linguistically appropriate, family-based lifestyle intervention conducted with 171 individuals of South Asian origin residing in the UK, the mean weight loss in the intervention group was 1.13 kg (SD 4.12) over 3 years and a mean weight gain of 0.51 kg (SD 3.65) in the control group [34].

Apart from modest changes in anthropometric parameters, a recent systematic review noted that most culturally and linguistically appropriate interventions of nutrition education conducted with the South Asian population have mixed effects on clinical parameters [15]. However, to our knowledge, there has been no similar intervention that has comprehensively measured the effect of these interventions on the dietary knowledge and skills of immigrants of South Asian origin or specifically immigrant women of Pakistani origin.

To assess improvements in nutritional knowledge and skills, due to the lack of validated tools, ad hoc questionnaires were used, complemented by FGDs, in order to obtain more comprehensive information. Our intervention significantly enhanced the nutritional knowledge and skills of participants from both the control and the intervention group with the intervention group exhibiting greater improvements (Table 3, Fig 2).

A probable reason for this considerable improvement might be Pakistani women’s pre-existing nutritional abilities, which stem from their primary role in managing dietary aspects at the household level. Assuming this solid foundation in nutrition knowledge and skills, even modest enhancement in these abilities may have led to significant improvements. These findings were reinforced by pre-intervention FGD since participants had adequate knowledge of the basics of healthy eating and appropriate cooking skills at baseline [30]. During the post-intervention FGD, a significant improvement in nutrition knowledge and skills was observed.

Despite the lack of studies specifically evaluating improvements in nutritional knowledge in the immigrant population of Pakistani origin, a systematic review conducted in the United States evaluated nine culturally and linguistically adapted studies carried out in other Asian groups and found improvements in their nutritional knowledge [35].

To assess the changes in dietary patterns, due to the susceptibility to memory bias of FFQ, data was combined with FGDs. Both methods confirmed significant improvements in the dietary patterns of the participants from both the control and the intervention groups. These improvements consisted of an increase in the consumption of vegetables, fruits, legumes, and nuts and a reduction in the intake of processed and ultra-processed products. Similar results were found in the InnvaDiab-DE-PLAN study, in which the results from the FFQ indicated an increase in the daily intake of vegetables, fruits, and fruit juices and a reduction in the consumption of red meats, full-fat milk/yogurt, and sugar-rich drinks in Pakistani women residing in Norway [17]. However, due to the high content of free sugars and low fiber, the intake of fruit juices was not evaluated positively in our study.

Triches and Giugliani [36] establish that individual nutrition knowledge might promote healthy food consumption and hence encourage changes in dietary behaviors. However, in a systematic review, Spronk et al. [37] illustrated this relationship as complex exhibiting weak associations. Conversely, our findings demonstrate notable improvements in both domains, indicating a robust relationship between knowledge and behavior in immigrant women of Pakistani origin. Furthermore, the control group, despite receiving three sessions, demonstrated significant improvement across all study variables, particularly in nutrition knowledge, skills, and dietary patterns. This indicates that even brief food education interventions can be effective for immigrant women of Pakistani origin while co-creating with them and adapting to their cultural and linguistic needs. However, it is essential to acknowledge, that in addition to nutrition knowledge and skills, several factors, including economic limitations and food accessibility, can also influence dietary decisions.

Our study differs from previous research conducted with South Asian or immigrant women of Pakistani origin through its methodological approach and outcome measures. Firstly, our intervention was co-created with the participants employing CBPR research principles. Secondly, we utilized a mixed-methods approach to complement and compare the qualitative and quantitative data. Thirdly, each session was based on a phase of the Transtheoretical model of behavior change, which contributed to personalizing and adapting nutritional advice and dynamics to the study population. Lastly, we extended beyond BMI and biochemical parameters to assess improvements in the participants’ nutrition knowledge, skills, and dietary patterns.

The main limitation of our study is the absence of clinical parameters, which restricts a comprehensive analysis that could reinforce the validity of our results. Furthermore, due to the lack of validated measuring instruments related to the dietary and nutritional aspects of the study population, we had to utilize ad hoc questionnaires and complement this information with a qualitative method. Future research should focus on developing validated measurement tools for immigrants of Pakistani origin and include assessments of both clinical and behavioral change parameters to enhance the robustness of the results. Another limitation of the study is the limited sample size, which restricts the generalizability of the findings.

## Conclusions

This study demonstrated that a culturally and linguistically appropriate food education program–the PakCat Program is effective in improving the nutrition status, nutrition knowledge and skills, and dietary pattern of immigrant women of Pakistani origin residing in Catalonia. However, to confirm the effectiveness of the proposed intervention, future studies should incorporate a larger sample across diverse geographical areas and combine the behavioral and clinical parameters. The development and validation of measuring instruments for behavioral changes is also necessary to facilitate a rapid and efficient replication of the intervention.

## Supporting information

S1 Checklist CONSORT 2010 checklist of information to include when reporting a randomised trial*. (DOC)

S1 File Original protocol (Catalan). (PDF)

S2 File Translated protocol (English). (PDF)

S3 File Questionnaires (Catalan English). (PDF)

S4 File Scoring criteria for Food Frequency Questionnaire (FFQ). (PDF)
