# Supplementary material for: Effect of the PakCat program on nutrition status, dietary pattern and nutrition knowledge and skills of Pakistani women living in Catalonia evaluated by a mixed-method randomized control trial (RCT)
Source: PLoS One. 2025 Jan 14;20(1):e0316803. doi: 10.1371/journal.pone.0316803 (PMC11731702; doi:10.1371/journal.pone.0316803)
Supplement: S1 File — (PDF) [file pone.0316803.s002.pdf]

DONES PAKISTANESES: AGENTS PROMOTORES D'HÀBITS ALIMENTARIS SALUDABLES

SABA ANWAR

**Director/a**	**Tutora**
Dra. Cristina Vaqué Crusellas	Dra. Cristina Larrea Killinger
Dr. Jesus Contreras Hernández	

1) Antecedents i estat actual del tema

1.1 Antecedents

El patró dietètic tradicional de la població pakistanesa es basa en el consum d'aliments majoritàriament d'origen vegetal, mínimament processats (aliments pertanyents al grup 1 d'acord amb la classificació NOVA)[1], frescs, de temporada i conreats localment. A més, pertanyent a un país en vies de desenvolupament on l'avenç tecnològic i infraestructural encara és limitat, aquest patró alimentari sol vincular-se a la pràctica d'un estil de vida moderadament actiu. Tanmateix, quan aquesta població emigra als països desenvolupats i adapta el patró dietètic occidental (elevat consum de carn, productes processats amb elevat contingut de sucre i greixos saturats i trans) realitzant també canvis en el seu estil de vida, augmenta el risc de patir algunes patologies metabòliques com ara la diabetis mellitus tipus 2 i l'obesitat.

La susceptibilitat dels immigrants sud-asiàtics (pakistanesos, indis i bangladeshis) a patir diabetis mellitus tipus 2 va ser establerta, per primera vegada al Regne Unit durant l'any 1985[2]. Poc després, a l'any 1989 es va trobar que la prevalença de diverses cardiopaties també és més elevada en els immigrants d'origen sud-asiàtic comparat amb la població europea[3]. Aquests resultats van obrir la porta a moltes investigacions per determinar l'estat de salut d'aquest col·lectiu. Al començament del segle XXI, es va establir que el risc de desenvolupar el síndrome metabòlic que engloba els factors de risc cardiovasculars (obesitat central, pressió arterial elevada, nivells elevats de glucosa plasmàtica i dislipèmia), així com les seves complicacions és més elevat en els immigrants d'Àsia Meridional en comparació amb la resta d'ètnies[4-5-6].

1.1 Estat actual del tema

Actualment, Pakistan és el cinquè país més poblat del món amb 220 milions d'habitants[7], dels quals 8,8 milions viuen a l'estranger[8]. L'Orient Mitjà, el Regne Unit, l'Europa i els Estat Units d'Amèrica són els destins principals dels immigrants pakistanesos[8]. A Espanya, el moviment migratori pakistanès es va iniciar als anys setanta quan el Regne Unit, el seu destí preferit de tots els temps, va restringir les polítiques d'immigració[9]. Tot i així, el creixement més gran es va produir a partir de l'any 2001 causada per la flexibilitat en els processos de regularització dels immigrants[9].

Segons l'Institut Nacional d'Estadística (2020), actualment el número de pakistanesos residents a Espanya és 97.705 , dels quals el 56 % viuen a Catalunya[10-11]. Segons l'Institut d'Estadística de Catalunya (2020) Pakistan és la cinquena població estrangera resident a Catalunya amb 54.571 habitants[10-11]. En quan al lloc de residència, la majoria de la població (87,9%) es troba resident a la província de Barcelona específicament a la comarca de Barcelonès (67,71%): Barcelona (61,51%), Badalona (18,01%), Hospitalet de Llobregat (11,09%), Santa Coloma de Gramenet (6,99%) i Sant Adrià de Besòs (2,40%)[10-12].

Tal com s'observa en la gràfica 1, la prevalença d'homes i de joves són dos elements característics de la població estrangera d'origen pakistanès resident a Catalunya. Les franges d'edats predominants són dels 20 als 39 anys mentre que la població major de 60 anys és molt minoritària. D'altra banda, s'observa que el grup de dones en aquesta comunitat és una minoria (29%)[12], arribades majoritàriament a Catalunya per a la reagrupació familiar pels seus marits o pares.

Gràfica 1. Diferències del sexe i edat en la població estrangera d'origen pakistanès resident a Catalunya

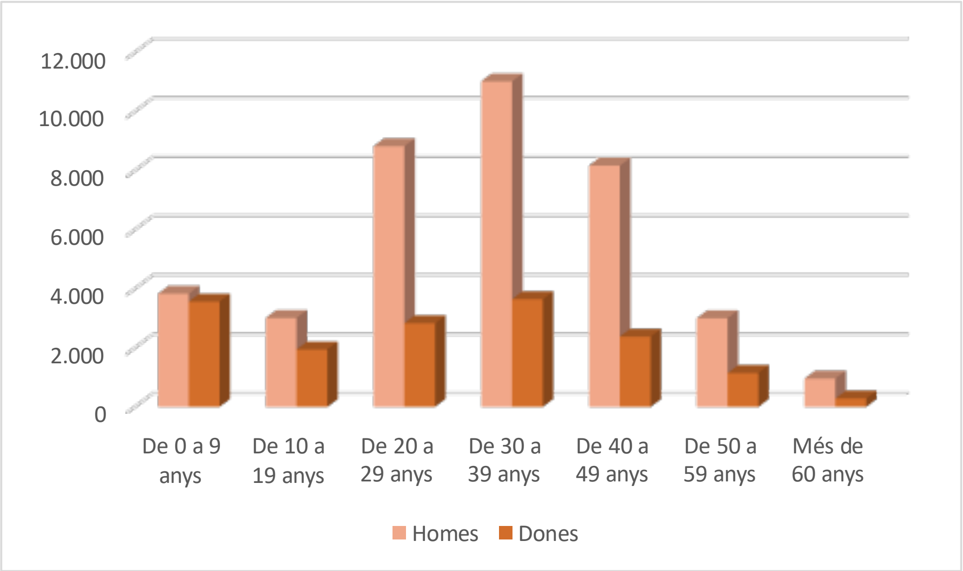

Font: Elaboració pròpia a partir de dades de l'Institut d'Estadística de Catalunya (Idescat)

Degut a les barreres culturals i lingüístiques juntament amb els obstacles per accedir al món laboral, les dones immigrades d'origen pakistanès són un dels col·lectius més invisible de Catalunya. Per donar-los veu, a l'any 2018 l'Ajuntament de Barcelona va realitzar un estudi "Barcelonines d'origen pakistanès" amb l'objectiu de conèixer el seu perfil socioeconòmic i permetre'ls l'expressió per dissenyar mecanismes d'apoderament a través de les seves propostes i demandes[33]. En aquest projecte les dones van proposar diferents

accions de promoció sociocultural i empoderament subjectiu i emocional. Aquestes propostes consistien en el disseny de projectes formatius per potenciar referents culturals, xerrades per fomentar la participació politicosocial i tallers d'educació emocional i treball personal.

Tot i que el perfil sociodemogràfic i socioeconòmic de la població pakistanesa resident a Catalunya és ben conegut, fins a l'actualitat, el context català no es disposa de cap informació sobre els seus aspectes de salut i alimentació. Tot i així, aquestes dades són disponibles als països amb un major número d'habitants pakistanesos com Noruega, Regne Unit, Estats Units i Austràlia. Degut a que els immigrants procedents de l'Índia, Pakistan i Bangladesh comparteixen el mateix perfil sociocultural i lingüístic[14], la recerca en aspectes de salut i alimentació, sovint es realitza de manera conjunta per a aquests tres col·lectius.

Actualment, s'estima que la prevalença del síndrome metabòlic en els immigrants d'Àsia Meridional és un 50% als Estats Units[15] i un 40 % al Regne Unit[16]. També es coneix que són la població més afectada per les malalties cardiovasculars i poden arribar a desenvolupar-les en edats molt més primerenques comparat amb qualsevol altra població[17-18]. Els components genètics i metabòlics[17-18], les elevades reserves del greix corporal[19], la baixa massa magra[20], el baix pes al nàixer[21-22], un patró dietètic poc saludable[14-15] i el sedentarisme[14-15] són les causes principals d'aquesta susceptibilitat.

A més a més, degut a les barreres culturals i lingüístiques els immigrants sud-asiàtics tampoc tendeixen a participar en els programes estàndards de promoció de salut. Les diferències en el comportament dietètic, les percepcions sobre la salut i la malaltia, així com les creences alimentàries que tenen, són altres factors que també poden obstaculitzar la comunicació amb el personal sanitari que intenten posar en pràctica els programes de promoció de la salut[22-23-24].

Per afrontar aquesta situació, diferents països han dissenyat i implementat els programes per promoure'n la salut cultural i lingüísticament adaptats a aquest col·lectiu que han resultat molt efectius. A la taula 1 es resumeixen alguns d'aquests programes dirigits als immigrants sud-asiàtics amb resultats satisfactoris pel que fa a la millora dels hàbits dietètics i l'estil de vida[25-26-27].

Taula 1. Intervencions culturals i lingüísticament adaptades a les dones pakistaneses amb l'objectiu de millorar el seu perfil metabòlic

INTERVENCIONS EDUCATIVES SOBRE UN ESTIL DE VIDA SALUDABLE PER ALS IMMIGRANTS SUDASIATICS									
Primer autor (any de publicació)	Disseny d'estudi	Criteris d'inclusió i exclusió	(N)	Grup exposició	Grup Control	Seguiment	Variables metabòlics	Dades inicials	Dades finals
Bhopal (2014)[25]	Prova controlada aleatòria (PCA)	***Inclusió***<br>- Immigrants d'origen Indi o pakistanès residents a Glascow o Edimburg, Escòcia<br>- ≥ 35 anys d'edat<br>- Circumferència de la cintura ≥ 90 cm homes o ≥ 80 cm dones<br>***Exclusió***<br>- DM tipus 2<br>- Embaràs<br>- Tractament actiu amb esteroides<br>- Ús de medicaments per reducció de pes<br>- Malaltia que impedeixin la participació a l'estudi	- Grup Intervenció (n=85)<br>- Grup control (n=86)<br>Total (n=171)	- 15 visites d'una dietista a les llars dels participants durant 3 anys.<br>- Cada dietista visitava a les mateixes famílies al llarg de 3 anys proporcionant la informació nutricional culturalment adaptada.<br>- Informació amb exemples sobre les compres i cocció dels aliments.<br>- Sessions anuals de compres alimentàries i caminar en grup.	- Quatre visites d'una dietista durant tres anys.<br>- Assessorament escrit i verbal estandarditzat per menjar més saludablement.	36 mesos	- Circumferència de la cintura (cm)<br>- PAS/PAD (mm Hg)<br>- Índex de massa corporal (kg/m2)<br>- Glucèmia basal (mmol/l)	102.7<br>137/83<br>30.6<br>5.8	100.5<br>137/81<br>30.2<br>5.8
Kandula (2015)[26]	Prova controlada aleatòria (PCA)	***Inclusió***<br>- Els immigrants sud asiàtics de l'Índia i Pakistan residents a Chicago, Estats Units.<br>- Rang d'edat entre 30 i 59 anys.<br>- Tenir com a mínim un factor de risc de malaltia cardiovascular ateroscleròtica<br>***Exclusió***<br>- Conviure a la mateixa llar	- Grup Intervenció (n=31)<br>- Grup control (n=32)<br>Total (n=63)	- 6 intervencions grupals sobre un estil de vida saludable cultural i lingüísticament adaptades.<br>- Al acabar les sessions, suport telefònic individualitzat durant 10 setmanes.<br>- 4 tallers de cuina i activitat física a l'aire lliure	- Enviament per el correu del materials educatiu traduït sobre les malalties cardiovasculars ateroscleròtiques i comportaments saludables.	6 mesos	- Circumferència de la cintura (cm)<br>- PAS (mm Hg)<br>- Glucèmia basal (mgl/dl)	95<br>127<br>109	94.3<br>123.4<br>107.2
**INTERVENCIONS EDUCATIVES SOBRE UN ESTIL DE VIDA SALUDABLE PER A LES DONES PAKISTANESES**									
Primer autor (any de publicació)	Disseny d'estudi	Criteris d'inclusió i exclusió	(N)	Grup exposició	Grup Control	Seguiment	Variables metabòlics	Dades inicials	Dades finals
Telle-Hjellset(2012)[32]	Prova controlada aleatòria (PCA)	***Inclusió***<br>- Dones pakistaneses residents a Noruega<br>- Progenitors pakistanesos<br>- ≥ 25 anys d'edat<br>***Exclusió***<br>- Embaràs<br>- Diabetis Mellitus tipus 2<br>- Malalties cardiovasculars<br>- Tractament actiu d'hipertensió arterial	- Grup Intervenció (n=101)<br>- Grup control (n=97)<br>Total (n=198)	- 6 sessions d'educació alimentària cultural i lingüísticament adaptades a les dones participants.<br>- *Feedback* individual sobre l'analítica de sang<br>- Animar a Caminar en grup per 1h durant dos dies a la setmana<br>- Facilitar sabates adequades per caminar i serveis de guarderia<br>- Indicació de llocs adequats per caminar amb cotxets per a nadons	- 1 sessió sobre l'estil de vida saludable<br>-*Feedback* individual sobre l'analítica de sang	7 mesos	- Circumferència de la cintura (cm)<br>- PAS/PAD (mm Hg)<br>- Glucèmia basal (mmol/l)<br>- HDL (mmol/l)<br>-Triglicèrids (mmol/l)<br>- Síndrome metabòlic (%)	95.3<br>116/80<br>5.6<br>1.26<br>1.4<br>44	95.1<br>114/79<br>5.4<br>1.24<br>1.3<br>42
Kousar (2008)[23]	Estudi de cohort (un grup pre i post intervenció)	***Inclusió***<br>- Dones pakistaneses residents a Melbourne amb un temps d'estada superior a 5 anys<br>- Rang d'edat entre 20-60<br>- Presentar com a mínim un component del síndrome metabòlic<br>***Exclusió***<br>-	Total (n=40)	- 4 hores d'assessorament setmanal durant 12 setmanes a càrrec d'un nutricionista bilingüe<br>- Material cultural i lingüísticament adaptat a les participants<br>- 1 mòdul setmanal d'educació alimentària (fomentar varietat, tipus de greix, etc)<br>- Objectius setmanals establerts i discutit els de la setmana anterior.<br>- Sessions realitzades a les pròpies llars dels participants incloent altres membres de família<br>- Instruccions de caminar 10.000 passos per sis vegades a la setmana	-	3 mesos	- Índex de massa corporal (kg/m2)<br>- Glucèmia basal (mmol/l)<br>- PA (mm Hg)<br>-Triglicèrids (mmol/l)	29.2<br>6.4<br>-<br>2.9	29.1<br>6.3<br>125/80<br>2.6

Dins el conjunt de la població sud-asiàtica, la prevalença del síndrome metabòlic i malalties cardiovasculars és més elevada en les dones que amb els homes, especialment en la població pakistanesa [22-29]. La taxa de mortalitat per les patologies mencionades també sol ser més alta en les dones pakistaneses que amb els homes[22-28]. A part de tenir una elevada susceptibilitat a aquestes patologies, les dones pakistaneses també solen afrontar més dificultats a l'hora d'integrar-se al país d'acollida comparat amb els homes del mateix origen[22-23-28]. Els hi afecta en major grau les barreres culturals i lingüístiques, com a conseqüència de l'estrès d'aculturació i l'aïllament social, comú entre elles[30].

Amb tot, la salut de les dones immigrades d'origen pakistanès, tant a nivell físic com psicològic es pot deteriorar al llarg dels anys de residència als països occidentals[22-30]. Per tant, en alguns països s'han dissenyat i implementat les intervencions alimentàries i d'estil de vida, cultural i lingüísticament apropiades, dirigides específicament a les dones pakistaneses. A la taula 1 també es destaquen algunes d'aquestes intervencions.

Un estudi que hi fa referència és l'estudi "The InnvaDiab study" realitzat pel Telle-Hjellset et al. (2012) que va tenir lloc a Oslo, Noruega. Aquest assaig controlat aleatori amb 198 participants (entre 25 i 62 anys d'edat) va tenir una durada de 7 mesos i es van realitzar 6 sessions educatives sobre el control glucèmic, la dieta i l'activitat física. Els components metabòlics a tenir en compte eren la circumferència de la cintura, la pressió arterial, la glucèmia basal, els triglicèrids i HDL, mesurats 1-3 setmanes abans i després de la intervenció. Pel que fa el seguiment d'un patró dietètic saludable, es va utilitzar el model Transteòric[31] per identificar en quina fase de motivació al canvi es troben les participants de cada grup. Aquest model concep el canvi de conducta com un procés gradual, dinàmic i continu, establint 5 etapes: (1) precontemplació: escassa consciència de la necessitat de canvi a curt termini, (2) contemplació: etapa d'ambivalència, consciència de la necessitat de canvi no prevista en un futur pròxim de 6 mesos, (3) preparació: plantejament de canvi en un futur proper, generalment en 1 mes, (4) acció: es duen a terme accions per canviar, i (5) manteniment: les accions es mantenen per més de 6 mesos.

A finalitzar l'estudi, es van observar millores importants en el perfil metabòlic juntament amb els hàbits dietètics i l'estil de vida de les participants que van formar part del grup intervenció. A més, la majoria de les dones pertanyents a aquest grup es situaven a la fase d'acció, per tant estaven portant a terme les accions per aconseguir la conducta desitjada.

En un altre estudi realitzat pel Kousar, Burns i Lewandowski (2008) que va tenir lloc a Melbourne, Austràlia, es va observar l'estil de vida saludable d'una cohort de 40 dones immigrades d'origen pakistanès residents a Melbourne. Es realitzava una sessió setmanal al llarg d'aproximadament 3 mesos a nivell familiar tot i que les dones eren la població principal de l'estudi. Es va controlar els components del síndrome metabòlic: IMC, glucèmia basal, pressió arterial i triglicèrids. Tot i que, degut a la curta durada de la intervenció, a curt termini, les millores en el perfil metabòlic no eres significatives, però al llarg termini, es van observar millores rellevants en els components del síndrome metabòlic en les dones participants.

En definitiva, la majoria de les intervencions educatives per promoure estils de vida saludables culturalment i lingüísticament adaptades han resultat efectives per millorar els hàbits alimentaris i el perfil metabòlic de les dones immigrants d'origen pakistanès en diferents regions del món. Seguint aquesta línia, en la present tesi doctoral es proposa un projecte similar per a les dones Pakistaneses residents a Catalunya.

2. Objectius i hipòtesis

2.1 Hipòtesi

La participació de les dones migrades d'origen pakistanès en un programa d'educació alimentària basat en el model d'etapes de canvi, cultural i lingüísticament adaptat, millorarà els seus hàbits i coneixements alimentaris capacitant-les per esdevenir agents promotors d'hàbits alimentaris saludable a la seva comunitat.

2.2 Objectius

2.2.1 Objectiu general

Avaluar l'eficàcia d'un programa d'educació alimentària basat en el model d'etapes de canvi, cultural i lingüísticament adaptat a les dones pakistaneses residents a Badalona i Santa Coloma de Gramenet.

2.2.2 Objectius específics

- Descriure la dieta de les dones participants.
- Identificar les fortaleses i debilitats del seu patró dietètic actual en comparació amb el tradicional.
- Definir l'estat nutricional i de la salut de la població objectiu.
- Explorar els coneixements i les creences culturals i religioses relacionades amb l'alimentació de la població objectiu.
- Dissenyar i implementar un programa d'educació alimentària cultural i lingüísticament adaptat a la població d'estudi.
- Incrementar els coneixements i habilitats dietètics de les participants.
- Millorar els seus hàbits alimentaris incrementant el consum de fruites i hortalisses, llegums i fruits secs, i reduint-ne del sal, sucre, carn vermella i processada i aliments ultraprocessats.
- Estudiar els canvis en la motivació pel que fa a les millores en el seu patró dietètic d'acord amb el model Transteòric.
- Determinar els canvis en la ingesta alimentària i l'estat nutricional de les participants al llarg de la intervenció.
- Avaluar l'adequació de la intervenció en quan a qüestions lingüístiques i culturals juntament amb el nivell de satisfacció dels participants.

3. Metodologia

3.1 Àmbit d'estudi

Aquest projecte es realitzarà a la província de Barcelona, específicament a la comarca de Barcelonès, que és la llar de més de la meitat de la població estrangera d'origen pakistanès resident a Catalunya[11]. En concret, aquest estudi tindrà lloc a Badalona i Santa Coloma de Gramenet per les raons que s'expliciten a continuació: són dos municipis veïns i respectivament, el segon i quart més poblat de Catalunya per la població pakistanesa[10] i els seus residents pakistanesos tenen un perfil sociodemogràfic i socioeconòmic força similar.

Les dones pakistaneses residents a aquests municipis compten amb el suport de dues institucions que comparteixen un mateix objectiu: integració de les persones migrades a la societat i l'entorn d'acollida.

A Badalona, des de la dècada dels anys 60, la Fundació Ateneu Sant Roc està treballant amb la missió d'afavorir la cohesió social del seu barri, promovent diversos projectes socioculturals i educatius dirigits a infants, joves i adults, però sobretot als col·lectius més vulnerables que es troben en risc d'exclusió social, entre elles destaquen les dones immigrades d'origen pakistanès. Actualment, la Fundació atén aproximadament a 70 dones pakistaneses d'edats diferents i els proporciona suport personalitzat, de manera que les joves pakistaneses hi reben l'acompanyament en els seus estudis acadèmics i les adultes aprenen el castellà, el català o dues i informàtica. Una gran part de les dones pakistaneses també estan vinculades als projectes de desenvolupament comunitari de la Fundació Ateneu Sant Roc, en els qual es realitzen diverses activitats de promoció sociocultural i educativa, i formació sociosanitària.

A Santa Coloma de Gramenet, Casa Àsia que és un centre cultural ubicat a Barcelona, durant l'any 2018 va iniciar el seu projecte "Aprenem. Famílies en Xarxa (AFEX)" a diferents centres educatius amb l'objectiu de promoure la integració de persones amb contextos culturals diversos a través d'aprenentatge de castellà, català i informàtica, amb l'ajuda dels seus propis fills i filles. Actualment, més de 50 dones pakistaneses residents a Santa Coloma de Gramenet formen part del projecte AFEX.

Les dues institucions han acceptat col·laborar en aquest estudi possibilitant que totes les dones d'origen pakistanès migrades a Barcelona, vinculades a aquestes institucions, puguin participar en el projecte.

3.2 Disseny i la població de l'estudi

En aquest estudi experimental controlat aleatòriament d'àmbit comunitari, hi podrà participar qualsevol dona pakistanesa vinculada a entitats col·laboradores sempre que compleixi els criteris d'inclusió establerts: ser major d'edat (>18 anys), tenir la residència a Badalona o Santa Coloma de Gramenet i acceptar voluntàriament la participació a l'estudi. Com a criteris d'exclusió s'han establert: convivència amb alguna participant, tenir el diagnòstic d'alguna patologia física o psíquica que impedeixi la seva participació al projecte o estar en desacord amb les condicions ètiques de l'estudi. Tenint en compte el número de dones que atenen les entitats col·laboradores s'ha decidit formar una mostra de 120 dones (60 a cada entitat).

L'estudi seguirà un disseny metodològic mixte, combinant models i tècniques d'obtenció d'informació i anàlisis de dades qualitatives i quantitatives.

3.3 La intervenció

El desenvolupament de l'estudi està previst en diferent fases:

- **Fase 0: Difusió i reclutament**

  Aquesta fase correspon a la difusió del projecte i reclutament de les institucions i participants, després d'obtenir l'aprovació de l'estudi pel Comissió de Bioètica. S'enviarà una carta de presentació a les dues entitats seleccionades per a la participació a l'estudi, presentant detalladament el projecte i demanant la seva col·laboració. Una vegada hagin acceptat la proposta de col·laboració, es formalitzarà el consentiment de participació voluntària de les institucions. Posteriorment podrem iniciar la difusió del projecte i reclutament de les participants que es concreta en trobades amb els educadors/es i voluntaris/es d'aquestes entitats, així com a les seves usuàries pakistaneses per donar a conèixer l'abast, els objectius i les condicions ètiques de l'estudi. Durant les trobades amb les dones pakistaneses, la comunicació serà efectuada en urdú i panjabi.

  A partir d'aquí, es realitzarà una nova trobada amb les usuàries que hagin mostrat interès per concretar detalls del projecte i les implicacions de la participació. A totes

les persones que vulguin participar-hi, se'ls entregarà el consentiment informat. Els educadors/es dels grups de les institucions col·laboradores seran els encarregats/des de recollir-los signats.

En endavant, tant l'equip com les usuàries pakistaneses de les entitats col·laboradores donaran suport en el procés de difusió del projecte i reclutament de participants, invitant a familiars o coneguts a participar-hi, fins formar els grups de dones que requereix el projecte.

- **Fase 1: Recopilació de dades**

  En aquesta fase exploratòria del projecte es recolliran les dades de les participants dels dos grups, control-exposició. Aquesta recopilació de dades serà abans de començar la intervenció (pre-intervenció) utilitzant la combinació de mètodes i tècniques quantitatives i qualitatives.

  Des de la perspectiva quantitativa, s'administrarà una enquesta per determinar les dades sociodemogràfiques, clíniques, antropomètriques i alimentàries. A partir de les dades sociodemogràfiques (edat, estudis, temps de residència a Catalunya, factors socioeconòmics,...) es construirà una mostra representativa de les participants a cada entitat.

  Des de la perspectiva qualitativa i basant-nos amb el mètode fenomenològic[34], es realitzaran les entrevistes semiestructurades amb les dones que formen part de la mostra representativa amb l'objectiu de determinar les creences culturals i religioses relacionades amb l'alimentació juntament amb les fortaleses i limitacions del seu patró dietètic actual. La informació obtinguda a través d'aquestes entrevistes servirà, en part, per ajustar la proposta formativa a les necessitats i fortaleses de les participants.

  A part de l'entrevista també es realitzarà una exposició Photovoice per visualitzar la gastronomia pakistanesa, de manera cada participant elaborarà un plat saludable amb els ingredients que tenen un significat important per ella.

  La recollida de dades es farà individualment i, si la situació de la pandèmia ho possibilita es realitzarà presencialment a una sala de cada una de les entitats, en un ambient relaxat.

- **Fase 2: Implementació de la intervenció educativa**

  En aquesta fase es posarà en pràctica l'estratègia d'educació alimentària basada en el model d'etapes de canvi del model Transteòric, de manera que a cada entitat col·laboradora es formaran dos grups principals de dones (control i exposició) que

posteriorment es dividiran en quatre subgrups de quinze dones. Els subgrups del grup exposició rebran 10 sessions formatives durant 3 mesos, mentre que als subgrups del grup control atendran 3 sessions.

Tenint en compte les qüestions culturals i lingüístiques, les sessions es realitzaran en urdú i panjabi. Tots els continguts dels materials educatius també seran traduïts a l'urdú. Durant aquestes sessions es fomentaran els hàbits alimentaris saludables tenint present el patró dietètic tradicional de la població de l'estudi. Es reforçaran els coneixements relacionats amb l'alimentació tenint en compte les creences alimentàries de les dones participants i es treballaran habilitats de les participants per posar en pràctica conductes alimentàries saludables.

A continuació es concreta en què consistirà la intervenció d'educació alimentària basada en el model Transteòric:

Taula 2. Intervenció d'educació alimentària basada en el model Transteòric

ETAPES DE CANVI	OBJECTIUS	PROCÉS DEL CANVI	ESTRATÈGIA DEL CANVI
PRE-CONTEMPLACIÓ	Augmentar la consciència del problema i estimulant la possibilitat de canvi	*Consciousness raising*	Exposar i dialogar els canvis alimentaris i d'estil de vida degut al factor migratori.
		Dramatic relief	Presentar les dades sobre la prevalença del síndrome metabòlic i malalties cardiovasculars en els immigrants sud-asiàtics.
		Self-reevaluation	Grup focal sobre les fortaleses i debilitats del patró dietètic.
			Debat sobre les mites i creences relacionades amb l'alimentació i salut.
		Environmental reevaluation	Destacar l'impacte dels seus hàbits alimentaris a les generacions posteriors
CONTEMPLACIÓ	Aconseguir decantar la balança cap al canvi	*Consciousness raising*	Exposar els beneficis de seguir una alimentació saludable.
			Presentar els perjudicis de seguir una alimentació poc saludable.
		Self-reevaluation	Auto-avaluació dels hàbits alimentaris actuals mitjançant l'elaboració d'una llista dels hàbits saludables i malsans.
PREPARACIÓ	Reforçar els coneixements per facilitar el canvi.	*Countercondition ing*	Realitzar tallers sobre els aliments o conductes que cal potenciar, reduir o canviar-ne el tipus o qualitat, per portar a terme una alimentació saludable.
		Self-liberation	Recollir les propostes per adquirir els canvis aconsellats.
ACCIÓ	Efectuar el canvi potenciant l'autonomia	*Stimulus or environmental control*	Tallers sobre la planificació dietètica i la compra dels aliments; com elaborar plats saludables amb els aliments tradicionals; interpretació del etiquetatge nutricional, elaboració d'esmorzars i berenars saludables.
MANTENIMENT	Mantenir el canvi	*Helping relationships*	Exposició *Photovoice* dels plats saludables.
		Social liberation	Adquisició del rol d'agent promotors de salut per la resta de comunitat pakistanesa.

- **Fase 3: Avaluació**

  Al finalitzar totes les sessions d'educació alimentària, es realitzarà un qüestionari per identificar si hi ha hagut alguna modificació respecte les variables que s'han controlat a l'inici de l'estudi.

  Al cap de 3 mesos (curt termini) es repetirà l'avaluació, en la qual les dones es tornaran a realitzar l'enquesta i el quadern setmanal. També es prendran les mesures antropomètriques. Al cap de 6 mesos tindrà lloc l'avaluació a mig termini repetint el mateix procés que l'avaluació a curt termini.

3.2 Variables i els mètodes de mesura

Les variables i els mètodes se mesura seran les següents:

- **Edat**

  Es demanarà l'edat per identificar les diferències i similituds entre diferents variables.
- **Lloc de naixement**

  Es preguntarà el lloc de naixement determinant també si les dones participants van nàixer a zones urbanes o rurals.
- **Estat civil**

  Les opcions per determinar l'estat civil seran les següents: soltera, casada, divorciada o viuda.
- **Estudis acadèmics**

  Les categories que s'han per determinarà el nivell d'estudi són les següents: analfabetisme, educació infantil o equivalent, educació primària o equivalent, educació secundària o equivalent, formació professional o grau mitjà, batxillerat o grau superior i educació superior (grau, màster i/o doctorat). També es demanarà el país on hagin cursat els seus estudis.
- **Ocupació laboral**

  Les categories per determinar l'ocupació laboral són les següents: ocupada, desocupada o atur. S'identificaran les ocupacions tant al país d'origen com al país d'acollida. També es determinarà la professió de les dones participants.
- **Idiomes**

  Es demanarà quins idiomes parlen tant les dones participants com els seus familiars.

- **Religió**

    S'identificarà la religió de les dones participants.

- **Motiu de la migració**

    En l'enquesta es donaran 4 opcions (reagrupació familiar, estudis, motius laboral, altres) per determinar el motiu de la migració.

- **Anys de residència a Catalunya**

    Les dones diran el número d'anys que porten vivint a Catalunya.

- **Unitat familiar**

    Es demanarà la informació sobre els membres que formen part de la unitat familiar i les seves ocupacions.

- **Factors socioeconòmics**

    Els ingressos mensuals a nivell familiar juntament amb les ocupacions de diferents membres de la família ajudaran a determinar els factors socioeconòmics de la població.

- **Estat de salut**

    Per determinar l'estat de salut es demanarà si han tingut o actualment tenen alguna patologia. Es preguntarà concretament per les patologies més comunes entre la població pakistanesa com els components del síndrome metabòlic i malalties cardiovasculars. Es demanarà fins a quin grau necessiten prendre alguna medicació per seguir amb la seva rutina diària. Per últim, també es preguntarà si el factor migratori ha impactat la salut de les participants i com defineixen la seva salut actual.

- **Estat nutricional**

    L'estat nutricional es determinarà a través de les mesures antropomètriques. L'índex de massa corporal (IMC) calculat a partir del pes (kg) i l'alçada (cm) permetrà conèixer l'estat nutricional de les participants. S'utilitzarà la classificació de la Sociedad Española de Obesidad (SEEDO) per els valors de l'IMC per definir l'estat nutricional dels participants (taula 3). El valor normal de la circumferència de la cintura per a les dones sud-asiàtiques és ≥ 80 cm.

Taula 3. Valors de l'IMC

**< 18,5**	**Pes insuficient**
**18,5 – 24,9**	Normopès
**25 – 26,9**	Sobrepès Grau I
**27 – 29,9**	Sobrepès Grau II (pre-obesitat)
**30 – 34,9**	Obesitat Grau 1
**35 – 39,9**	Obesitat Grau 2
**40 – 49,9**	Obesitat Grau 3 (mòrbida)
**≥ 50**	Obesitat Grau 4 (extrema)

Font: Elaboració pròpia a partir de les dades de SEEDO.

- **Rol en temes d'alimentació**

  S'estudiarà el rol en temes d'alimentació identificant la responsabilitat de cuinar, planificar i realitzar les compres alimentàries i prendre la decisió de triar el menú. Es demanarà si les dones perceben que el fet de cuinar per la família és responsabilitat seva. Amb una escala de 10 (sent 0 menys important i 10 més important) s'estudiaran els factors que influeixen a l'hora de decidir el menú (cost, temporada, preferències de la família, etc)

- **Coneixements dietètics**

  Els coneixements dietètics es determinaran a partir de 14 afirmacions relacionades amb alguns aspectes de l'alimentació amb tres possibles respostes: cert, fals i no ho sé.

- **Habilitats dietètics**

  Les habilitats dietètics s'estudiaran a partir de 13 preguntes en referència als dificultats que han afrontat per a realitzar diferents accions en aspectes d'alimentació (planificació, compra, elaboració, etc dels aliments) durant els últims

  15 dies. Les respostes es recolliran amb 5 opcions: 1=Cap dificultat, 2= Poca dificultat, 3= alguna dificultat, 4= Força dificultat, 5= molta dificultat.

- **Patró dietètic**

  El patró dietètic s'estudiarà a través de l'enquesta, en el qual es determinaran els hàbits alimentaris especificant l'horari, el lloc i el número dels àpats diaris. També es demanarà de contestar un qüestionari de freqüència de consum alimentari inspirat en la Taula de Freqüències orientatives de la Sociedad Española de la Nutrición Comunitaria (SENC)[35]. Per complementar i profunditzar la informació obtinguda les participants ompliran un registre alimentari setmanal en el qual

especificaran el número, el lloc i l'horari dels àpats especificant també el tipus i la quantitat dels aliments ingerits al llarg d'una setmana.

- **Fortaleses i debilitats del patró dietètic**

  A través de les entrevistes es coneixeran les diferències entre el seu patró dietètic tradicional i actual causades per la immigració. També es descriuran els factors que les faciliten i dificulten el seguiment d'una alimentació saludable.

- **Creences alimentaries**

  A través de l'entrevista es determinaran les creences relacionades amb el consum de diferents aliments, els seus efectes a la salut i benestar, l'opinió sobre els productes naturistes, els mites relacionats amb l'alimentació i la salut, etc.

- **Millores alimentàries**

  Les millores alimentàries s'estudiaran a través d'un qüestionari que es realitzarà durant la fase d'avaluació, en el qual per cada aliment treballat durant les sessions es farà una pregunta amb cinc respostes possibles: (1) Pre-contemplació: "No he canviat el meu comportament durant els darrers 6 mesos i tampoc tinc previst fer-ho durant els propers 6 mesos." (2) Contemplació: "No he canviat el meu comportament durant els darrers 6 mesos, però estic pensant a fer-ho en els propers 6 mesos." (3) Preparació: "Actualment estic intentant canviar el meu comportament, però no de manera regular." (4) Acció: "Durant els darrers 6 mesos, ja he canviat el meu comportament." (5) Manteniment: "Vaig canviar el meu comportament fa 6 mesos."

- **Adequació cultural i lingüística**

  Al finalitzar les sessions les participants contestaran un qüestionari de satisfacció traduït en urdú sobre les qüestions lingüístiques, culturals i comprensió dels continguts.

4. Aspectes ètics

En aquesta intervenció es respectaran els aspectes ètics establerts establerts com el setè principi general de la Declaració de Hèlsinki de l'Associació Mèdica Mundial (AMM), de manera que abans de començar el projecte es realitzarà una primera visita a les usuaries pakistaneses de totes dues institucions. En aquesta visita s'explicarà detelladament tot el projecte en urdú. Per garantir la voluntat i la comprensió de les condicions de participació, tant el consentiment informat com el full de presentació s'ha traduït en urdú. Només hi podran participar les dones que hagin firmat el consentiment infromat.

Durant el procés de recollida, anàlisi i publicació de les dades es mantindrà l'anonimitat de les dones participants. Tota la informació obtinguda serà estrictament confidencial. La intervenció s'iniciarà després d'obtenir l'aprovació pel Comitè d'Ètica.

5. Aplicabilitat del projecte

Donat que les dones pakistaneses són referents a les seves famílies per els aspectes de salut i alimentació, al rebre l'educació alimentària cultural i lingüísticament adaptada, es capacitaran per poder extendre als seus familiars amb la finalitat d'obtenir un impacte en els hàbits alimentaris de les generacions posteriors. A més, convertir-se en agents promotores de salut permetrà contribuir a reforçar aspectes nutricionals i de salut de tota la seva comunitat fent sostenible la continuïtat de la intervenció. El fet que les dones portin aquesta iniciativa de promocionar hàbits alimentaris saludables a nivell familiar i comunitari, els atorgarà cert protagonisme social i familiar que contribuirà en el seu benestar personal afectat sovint per l'estrès d'aculturació que viuen aquestes dones.

Les sessions d'educació alimentària es realitzaran a nivell grupal, per tant l'aprenentatge serà cooperatiu i el reforç del grup ajudarà en potenciar les millores en els hàbits alimentaris de les dones participants. A més, aquesta pràctica fomentarà la socialització entre les dones pakistaneses que degut a les barreres culturals i lingüístiques, sovint se senten aïllades dins la societat d'acollida.

Aquesta estudi destacarà els problemes sociosanitaris més comuns d'aquest grup ètnic que fins el dia d'avui no han sigut explorats a Catalunya. Una implementació reeixida del programa, a part d'obrir futures línies d'investigació en camp de salut i alimentació per la població pakistanesa, també pot servir de model per crear intervencions de promoció d'hàbits alimentaris saludables cultural i lingüísticament adaptades per altres grups minoritaris.

6. Bibliografia

1. Monteiro CA, Cannon G, Levy RB et al. NOVA. The star shines bright. [Food classification. Public health] *World Nutrition* January-March 2016, 7, 1-3, 28-38.
2. Mather, H. M. & Keen, H. (1985) 'The Southhall Diabetes Survey: prevalence of known diabetes in Asians and Europeans', *British Medical Journal* , vol. 291, pp. 1081/1084.
3. McKeigue, P. M., Miller, G. J. & Marmot, M. G. (1989) 'Coronary heart disease in South Asians overseas: a review', *Journal of Clinical Epidemiology*, vol. 42, pp. 597/609.
4. Tillin T, Forouhi N, Johnston DG, McKeigue PM, Chaturvedi N, Godsland IF. Metabolic syndrome and coronary heart disease in South Asians, African-Caribbeans and white Europeans: a UK populationbased cross-sectional study. Diabetologia 2005;48:649-56.
5. Singh, R. B., Niaz, M. A., Ghosh, S., Beegom, R., Agarwal, P., Nangia, S., Moshiri, M. & Janus, E. D. (1998) 'Low fat intake and coronary artery disease in a population with higher prevalence of coronary artery disease: The Indian Paradox', Journal of the American College of Nutrition , vol. 17, pp. 342/350.
6. Farooqi A, Nagra D, Edgar T, Khunti K. Attitudes to lifestyle risk factors for coronary heart disease amongst South Asians in Leicester: a focus group study*. Fam Pract*. 2000 Aug;17(4):293-7.
7. United Nations, Department of Economic and Social Affairs, Population Division (2019). World Population Prospects 2019, Edició online. Rev. 1.
8. Ministeri de pakistanesos d'ultramar i desenvolupament de recursos humans. (2017-18). Year Book. Recuperat de http://www.ophrd.gov.pk/
9. Beltrán J, Sáiz A. La comunidad pakistaní en España. Anu Asia Pacífico CIDOB. 2007;407–16.
10. Institut Nacional d'Estadística. (2020). Población extranjera por país de nacionalidad, edad (grupos quinquenales) y sexo. Recuperat de https://www.ine.es/jaxiT3/Datos.htm?t=36825#!tabs-tabla
11. Institut d'estadística de Catalunya. (2020). Població estrangera per països. Recuperat de https://www.idescat.cat/poblacioestrangera/?b=12
12. Institut d'estadística de Catalunya. (2020). Població estrangera a 1 de gener. Per sexe i edat quinquennal. Recuperat de https://www.idescat.cat/poblacioestrangera/?geo=cat&nac=d426&b=1

13. Hierro-Olavarria, M. A. (2018). *Inmigración y acceso a los servicios públicos: la perspectiva del usuario: Estudio de la problemática comunicativa en el acceso a los servicios públicos de las mujeres pakistaníes de Barcelona.*Tesi doctoral. Universitat Autònoma de Barcelona, Catalunya.
14. Mellin-Olsen T, Wandel M. Changes in food habits among Pakistani immigrant women in Oslo, Norway. Ethnicity and Health 2005;10:311-39.
15. Khan SA, Jackson RT. The prevalence of metabolic syndrome among low-income South Asian Americans. *Public Health Nutr.* 2016;19:418–28.
16. Dodani S, Henkhaus R, Wick J, Vacek J, Gupta K, Dong L, Butler MG. Metabolic syndrome in South Asian immigrants: more than low HDL requiring aggressive management. *Lipids Health* Dis. 2011;10:45.
17. Kanaya AM, Herrington D, Vittinghoff E, Ewing SK, Liu K, Blaha MJ, Dave SS, Qureshi F, Kandula NR. Understanding the high prevalence of diabetes in U.S. south Asians compared with four racial/ethnic groups: the MASALA and MESA studies. *Diabetes Care.* 2014;37:1621–8.
18. Aryal, N., & Wasti, S. P. (2016). The prevalence of metabolic syndrome in South Asia: a systematic review. *International Journal of Diabetes in Developing Countries*, 36(3), 255-262. https://doi.org/10.1007/s13410-015-0365-5
19. U.P. Gujral, E. Vittinghoff, M. Mongraw-Chaffin, D. Vaidya, N.R. Kandula, M. Allison, J. Carr, K. Liu, K.M.V. Narayan, A.M. Kanaya, Cardiometabolic abnormalities among Normal-weight persons from five racial/ethnic groups in the United States: a cross-sectional analysis of two cohort studies, *Annals of Internal Medicine*. 166 (9) (2017) 628–636.
20. Gulati S, Misra A (2017) Abdominal obesity and type 2 diabetes in Asian Indians: dietary strategies including edible oils, cooking practices and sugar intake. European Journal of Clinical Nutrition 71(7):850–857. https://doi. org/10.1038/ejcn.2017.92
21. Krishnaveni GV, Yajnik CS (2017) Developmental origins of diabetes-an Indian perspective. European Journal of Clinical Nutrition .71(7):865–869. https://doi.org/10.1038/ejcn.2017.87
22. Mellin-Olsen T, Wandel M. Changes in food habits among Pakistani immigrant women in Oslo, Norway. Ethnicity and Health 2005;10:311-39.
23. Kousar, R., Burns, C., & Lewandowski, P. (2008). A culturally appropriate diet and lifestyle intervention can successfully treat the components of metabolic syndrome

in female Pakistani immigrants residing in Melbourne, Australia. *Metabolism: Clinical and Experimental*, 57(11), 1502–1508.

24. Choudhry, U. K., Jandu, S., Mahal, J., Singh, R., Sohi-Pabla, H., & Mutta, B. (2002). Health Promotion and Participatory Action Research with South Asian Women. *Journal of Nursing Scholarship, 34(1), 75–81.* doi:10.1111/j.1547-5069.2002.00075.x
25. Bhopal RS, Douglas A, Wallia S, Forbes JF, Lean ME, Gill JM, McKnight JA, Sattar N, Sheikh A, Wild SH, Tuomilehto J, Sharma A, Bhopal R, Smith JB, Butcher I, Murray GD. Effect of a lifestyle intervention on weight change in south Asian individuals in the UK at high risk of type 2 diabetes: a familycluster randomised controlled trial. *Lancet Diabetes Endocrinol.* 2014;2:218–27.
26. Kandula, N.R., Dave, S., De Chavez, P.J. et al. Translating a heart disease lifestyle intervention into the community: the South Asian Heart Lifestyle Intervention (SAHELI) study; a randomized control trial. *BMC Public Health* 15, 1064 (2015). https://doi.org/10.1186/s12889-015-2401-2
27. Jenum et al., (2019). Effects of dietary and physical activity interventions on the risk of type 2 diabetes in South Asians: meta-analysis of individual participant data from randomised controlled trials. *Diabetologia*. 62(8):1337-1348. doi: 10.1007/s00125-019-4905-2.
28. Johansen, K. S., Bjørge, B., Hjellset, V. T., Holmboe-, G., Wandel, M., & Ra, M. (2009). Changes in food habits and motivation for healthy eating among Pakistani women living in Norway : results from the InnvaDiab-DEPLAN study, 13(6), 858–867. https://doi.org/10.1017/S1368980009992047
29. Jafar, T. H., Levey, A. S., White, F. M., Gul, A., Jessani, S., Khan, A. Q., Chaturvedi, N. (2004). Ethnic differences and determinants of diabetes and central obesity among South Asians of Pakistan. Diabetic Medicine, 21(7), 716–723. doi:10.1111/j.1464-5491.2004.01140.x
30. Gask L, Aseem S, Waquas A, Waheed W. Isolation, feeling “stuck” and loss of control: Understanding persistence of depression in British Pakistani women. *Journal of affective Disorders*. 2011;128(1–2).
31. Prochaska, J.O.; Velicer, W.F. The transtheoretical model of health behavior change. Am. J. Heal. Promot. 1997, 12, 38–48, doi:10.4278/0890-1171-12.1.38.

32. Hjellset VT, Ihlebæk CM, Bjørge B, Eriksen HR, Høstmark AT. Health-Related Quality of Life, Subjective Health Complaints, Psychological Distress and Coping in Pakistani Immigrant Women With and Without the Metabolic Syndrome : The InnvaDiab-DEPLAN Study on Pakistani Immigrant Women Living in Oslo, Norway. J Immigr Minor Health. 2011 Aug;13(4):732-41. doi: 10.1007/s10903-010-9409-6.

33. Güell B, Martínez R, Naz K, Solé A. (2018). *Barcelonines d'origen pakistanès: empoderament i participació contra la feminització de la pobresa.* Ajuntament de Barcelona, Catalunya.

34. Høffding, S., Martiny, K. Framing a phenomenological interview: what, why and how. *Phenomenology and the Cognitive Sciences* 15, 539–564 (2016). https://doi.org/10.1007/s11097-015-9433-z

35. Sociedad Española de Nutrición Comunitaria. (2004). Guía de la alimentación saludable. Disponible a Sociedad Española De Nutrición Comunitaria (nutricioncomunitaria.org)
