# Supplementary material for: Effect of the PakCat program on nutrition status, dietary pattern and nutrition knowledge and skills of Pakistani women living in Catalonia evaluated by a mixed-method randomized control trial (RCT)
Source: PLoS One. 2025 Jan 14;20(1):e0316803. doi: 10.1371/journal.pone.0316803 (PMC11731702; doi:10.1371/journal.pone.0316803)
Supplement: S2 File — (PDF) [file pone.0316803.s003.pdf]

Pakistani Women: Promoting Agents of Healthy Eating Habits

SABA ANWAR

Directors
Dr. Cristina Vaqué Crusellas
Dr. Jesus Contreras Hernández

Tutor
Dr. Cristina Larrea Killinger

1) Background and Current Status of the Subject

1.1 Background

The traditional dietary pattern of the Pakistani population is based on the consumption of plant-based, minimally processed (foods belonging to group 1 according to the NOVA classification) [1], fresh, seasonal, and locally grown foods. In addition, belonging to a developing country where technological and infrastructural progress is still limited, this food pattern is usually linked to the practice of a moderately active lifestyle. However, when this population migrates to developed countries and adopts the Western dietary pattern (increasing the consumption of meat and processed products with a high content of sugar, saturated and trans fats), and makes changes in their lifestyle, the risk of suffering some metabolic diseases such as diabetes mellitus type 2 and obesity increases.

The susceptibility of South Asian immigrants (Pakistani, Indian, and Bangladeshis) to suffer from type 2 diabetes mellitus was established for the first time in the United Kingdom in 1985 [2]. Subsequently, in 1989, it was discovered that the prevalence of various heart diseases is also higher in immigrants of South Asian origin compared to the European population [3]. These results opened the door to many investigations conducted to determine the health status of this group. At the beginning of the 21st century, it was established that the risk of developing the metabolic syndrome, which includes cardiovascular risk factors (central obesity, high blood pressure, high plasma glucose levels, and dyslipidemia), as well as its complications, is higher in South Asian immigrants compared to other ethnic groups [4-5-6].

1.2 Current status of the subject

Currently, Pakistan is the fifth most populous country in the world with 220 million inhabitants [7], of which 8.8 million live abroad [8]. The Middle East, the United Kingdom, Europe, and the United States of America are the main destinations for immigrants of Pakistani origin [8]. Pakistani migration to Spain began in the 1970s when the United Kingdom, their favored destination, restricted immigration policies [9]. Nonetheless, the biggest expansion happened after 2001 as a result of the flexibility in immigration regularization processes [9].

According to the National Institute of Statistics (2020), currently, the number of Pakistanis resident in Spain is 97,705, of which 56% live in Catalonia [10-11]. According to the Statistical Institute of Catalonia (2020), Pakistan is the fifth foreign resident population in Catalonia with 54,571 inhabitants [10-11]. As for the place of residence, the majority of the population (87.9%) reside in the province of Barcelona, specifically in the region of Barcelonès (67.71%): Barcelona (61.51%), Badalona (18 .01%), Hospitalet de Llobregat (11.09%), Santa Coloma de Gramenet (6.99%) and Sant Adrià de Besòs (2.40%) [10-12].

As can be seen in Figure 1, the prevalence of men and young people are two characteristic elements of the foreign population of Pakistani origin residing in Catalonia. The predominant age groups are from 20 to 39 years while the population over 60 is a very small minority. it can also be observed that the group of women in this community is a minority (29%)[12], mostly arriving in Catalonia due to the family reunification procedures initiated by their husbands or fathers.

Due to the cultural and linguistic barriers along with obstacles to employment, immigrant women of Pakistani origin are one of the most invisible groups in Catalonia. To provide them a voice, the Barcelona City Council conducted research entitled "*Barcelonines d'origen pakistanès*" in 2018 to acquire knowledge about their socioeconomic status and allow them to express themselves so that empowerment mechanisms could be designed based on their suggestions and demands [33].

Figure 1. Gender and age differences in the foreign population of Pakistani origin residing in Catalonia

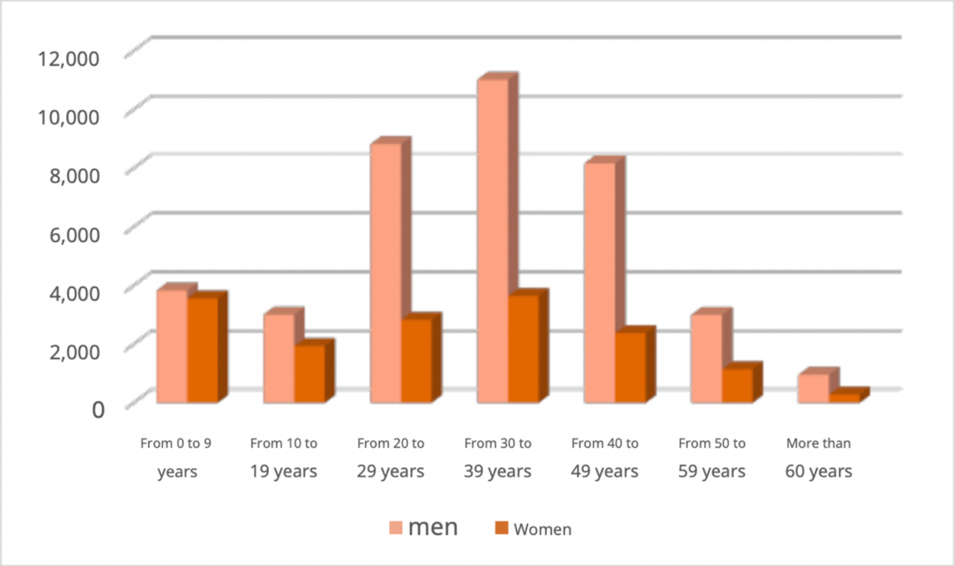

Source: Own elaboration based on data from the Institute of Statistics of Catalonia (Idescat)

In this initiative, the women presented several socio-cultural promotion actions, as well as their personal and emotional empowerment. These suggestions included the development of training initiatives to create cultural figures, interventions to promote political and social involvement, emotional education, and professional seminars.

Although the socio-demographic and socio-economic profile of the Pakistani population residing in Catalonia is well known, to date, the Catalan context does not have any information on its aspects of health and nutrition. However, these data are available in countries with the largest number of Pakistani inhabitants such as Norway, the United Kingdom, the United States, and Australia. As immigrants from India, Pakistan, and Bangladesh share the same socio-cultural and linguistic profile [14], research on aspects of health and nutrition is often carried out conjointly for these three groups.

Currently, it is estimated that the prevalence of metabolic syndrome in immigrants from South Asia is 50% in the United States [15] and 40% in the United Kingdom [16]. It is also known that they are the most affected population by cardiovascular diseases and may develop them at much younger ages compared to any other demographic [17-18]. Genetic and metabolic components [17-18], high body fat reserves [19], low lean mass [20], low birth weight [21-22], an unhealthy dietary pattern [14-15], and a sedentary lifestyle [14-15] are the main causes of this susceptibility. Furthermore, due to cultural and linguistic barriers, South Asian immigrants are less likely to participate in standard health promotion programs. Differences in dietary behavior, perceptions of health and illness, as well as dietary beliefs, are other factors that may also hinder communication with health personnel who try to implement health promotion programs [22-23-24].

To deal with this situation, different countries have designed and implemented culturally and linguistically adapted health promotion programs, which have been very effective. Table 1 summarizes some of these programs targeting South Asian immigrants with successful results in improving dietary habits and lifestyle [25-26-27].

Table 1. Culturally and linguistically adapted interventions for Pakistani women with the objective of improving their metabolic profile

First author (year of publication)	design of study	Inclusion and exclusion criteria	(N)	Intervention group	Control group	Follow-up	Metabolic variables	Initial data	Final data
**EDUCATIONAL INTERVENTIONS ON A HEALTHY LIFESTYLE FOR SOUTH ASIAN IMMIGRANTS**									
Bhopal (2014)[25]	Randomized Controlled Trial (RCT)	**Inclusion**<br>- Immigrants of Indian or Pakistani origin residing in Glasgow or Edinburgh, Scotland<br>- ≥ 35 years of age<br>- Waist circumference ≥ 90 cm for men or ≥ 80 cm for women<br>**Exclusion**<br>- DM type 2<br>- Pregnancy<br>- Active treatment with steroids<br>- Use of medications for weight reduction<br>- Illness that prevents participation in the study	- Intervention group (n=85)<br>- Control group (n=86)<br>Total (n=171)	- 15 visits by a dietitian to participants' homes over 3 years.<br>- Each dietitian visited the same families over the course of 3 years providing culturally adapted nutritional information.<br>- Information with examples about shopping and cooking food.<br>- Annual sessions of grocery shopping and group walking.	- Four visits by a dietician over three years.<br>- Standardized written and verbal advice to eat more healthily.	36 months	- Waist circumference (cm)<br>- PAS/PAD (mm Hg)<br>- Body mass index (kg/m^2)<br>- Fasting blood glucose(mmol/l)	102.7<br>137/83<br>30.6<br>5.8	100.5<br>137/81<br>30.2<br>5.8
Kandula (2015)[26]	Randomized Controlled Trial (RCT)	**Inclusion**<br>- South Asian immigrants from India and Pakistan residing in Chicago, United States.<br>- Age range between 30 and 59 years.<br>- Having at least one risk factor for atherosclerotic cardiovascular disease<br>**Exclusion**<br>- Living in the same household	- Intervention group (n=31)<br>- Control group (n=32)<br>Total (n=63)	- 6 culturally and linguistically adapted group interventions on a healthy lifestyle.<br>- At the end of the sessions, individualized telephone support for 10 weeks.<br>- 4 outdoor cooking and physical activity workshops	- Mailing of translated educational materials about atherosclerotic cardiovascular diseases and healthy behaviors.	6 months	-Waist circumference (cm)<br>- PAS (mm Hg)<br>- Fasting blood glucose(mmol/l)	95<br>127<br>109	94.3<br>123.4<br>107.2
**EDUCATIONAL INTERVENTIONS ON A HEALTHY LIFESTYLE FOR PAKISTANI WOMEN**									
First author (year of publication)	design of study	Inclusion and exclusion criteria	(N)	Intervention group	Control group	Follow-up	Metabolic variables	Initial data	Final data
Telle-Hjellset(2012)[32]	Randomized Controlled Trial (RCT)	**Inclusion**<br>- Pakistani women resident in Norway<br>- Pakistani parents<br>- ≥ 25 years of age<br>**Exclusion**<br>- Pregnancy<br>- Diabetes Mellitus type 2<br>- Cardiovascular diseases<br>- Active treatment of arterial hypertension	- Intervention group (n=101)<br>- Control group (n=97)<br>Total (n=198)	- 6 culturally and linguistically appropriate food education sessions<br>- Individual feedback on the blood analysis<br>- Encourage to walk in a group for 1 hour twice a week<br>- Provide suitable shoes for walking and childcare services<br>- Indication of suitable places for walking with baby carriage	- 1 session on healthy lifestyle<br>- Individual feedback on blood analysis	7 months	- Waist circumference (cm)<br>- PAS/PAD (mm Hg)<br>- Fasting blood glucose (mmol/l)<br>- HDL (mmol/l)<br>- Triglycerides (mmol/l)<br>-Metabolic syndrome (%)	95.3<br>116/80<br>5.6<br>1.26<br>1.4<br>44	95.1<br>114/79<br>5.4<br>1.24<br>1.3<br>42
Kousar (2008)[23]	Cohort study (a pre- and post-intervention group)	**Inclusion**<br>- Pakistani women resident in Melbourne with a stay of more than 5 years<br>- Age range between 20-60<br>- Present at least one component of the metabolic syndrome<br>**Exclusion**<br>-	Total (n=40)	- 4 hours of weekly counseling for 12 weeks by a bilingual nutritionist<br>- Cultural and linguistic material adapted to the participants<br>- 1 weekly food education module (encouraging variety, type of fat, etc.)<br>- Establishing weekly objectives and reviewing those from the previous week<br>- Sessions held in the participants' own homes including other family members<br>- Instructions to walk 10,000 steps six times a week	-	3 months	- Body mass index (kg/m^2)<br>- Fasting blood glucose(mmol/l)<br>- PAS (mm Hg)<br>- Triglycerides (mmol/l)	29.2<br>6.4<br>-<br>2.9	29.1<br>6.3<br>125/80<br>2.6

Cardiovascular disease and metabolic syndrome are more common in women than in males throughout the South Asian population, particularly in the Pakistani community [22–29]. The mortality rate from the mentioned diseases is also usually higher in Pakistani women than in men [22-28]. In addition to being more vulnerable to these diseases than males of the same origins, Pakistani women also experience more challenges integrating into the host country [22–23–28]. Cultural and linguistic barriers affect them to a greater degree, as a result of the stress of acculturation and social isolation, highly common among them [30].

Due to all these factors the physical and psychological health of migrant women of Pakistani origin deteriorates over the years of residence in Western countries [22-30]. To address this situation, some countries have successfully designed and implemented culturally and linguistically appropriate dietary and lifestyle interventions designed for Pakistani women. Table 1 also highlights some of these interventions.

One of these studies "The InnvaDiab study" was carried out by Telle-Hjellset et al. (2012) in Oslo, Norway. This randomized controlled trial conducted with 198 participants (between 25 and 62 years of age) lasted 7 months and included 6 educational sessions on glycemic control, diet, and physical activity. The metabolic components to consider were waist circumference, blood pressure, basal glycemia, triglycerides, and HDL, measured 1-3 weeks before and after the intervention. To monitor the dietary pattern, the Transtheoretical model [31] was utilized to determine the participants' motivational phase for change in each group. This model defines behavior modification as a progressive, dynamic, and continuous process, with five stages: (1) pre-contemplation: little awareness of the need for short-term change, (2) contemplation: stage of ambivalence, awareness of the need for change not expected in the near future of 6 months, (3) preparation: approach to change in the near future, usually within 1 month, (4) action: actions are taken to change, and (5) maintenance: the change is held for more than 6 months.

At the end of the study, significant improvements were observed in the metabolic profile along with the dietary habits and lifestyle of the participants who were part of the intervention group. In addition, the majority of the women belonging to this group were in the action phase, therefore they were carrying out the actions to achieve the desired behavior.

In another study by Kousar, Burns, and Lewandowski (2008) that took place in Melbourne, Australia, the lifestyle of a cohort of 40 Pakistani immigrant women living in Melbourne was observed. A weekly session was conducted over approximately 3 months at the family level, however women were the main study population. The components of the metabolic syndrome were monitored: BMI, basal glycemia, blood pressure, and triglycerides. Although the short duration of the intervention resulted in no significant improvements in the metabolic profile in the short term, substantial improvements in the metabolic syndrome components were identified in the female participants in the long term.

In short, the majority of the culturally and linguistically adapted educational interventions aimed to promote healthy lifestyles have been effective in improving the eating habits and metabolic profile of immigrant women of Pakistani origin in different regions of the world. Following this line, in this doctoral thesis, a similar project is proposed for Pakistani women resident in Catalonia.

2. Objectives and hypotheses

2.1 Hypothesis

The participation of migrant women of Pakistani origin in a culturally and linguistically adapted food education program based on the model of stages of change will improve their food habits and knowledge and empower them to become promoting agents of healthy eating habits in their community.

2.2 Objectives

2.2.1 General objective

To evaluate the effectiveness of a culturally and linguistically adapted food education program based on the model of stages of change for Pakistani women living in Badalona and Santa Coloma de Gramenet.

2.2.2 Specific objectives

- Describe the diet of the participating women.
- Identify the strengths and weaknesses of their current dietary pattern compared to the traditional one.
- Define the nutritional and health status of the target population.
- Explore the food-related cultural and religious beliefs and knowledge of the target population.
- Design and implement a culturally and linguistically appropriate food education program for the study population.
- Increase the dietary knowledge and skills of the participants.
- Improve their eating habits by increasing the consumption of fruit and vegetables, legumes and nuts, and reducing salt, sugar, red and processed meat, and ultra-processed foods.
- Study changes in motivation regarding improvements in their dietary pattern according to the Transtheoretical model.
- Determine the changes in food intake and nutritional status of the participants throughout the intervention.
- Assess the adequacy of the intervention in terms of linguistic and cultural issues along with the level of satisfaction of the participants.

3. Methodology

3.1 Study setting

This project will be carried out in the province of Barcelona, specifically in the region of Barcelonès, which is home to more than half of the foreign population of Pakistani origin residing in Catalonia [11]. Specifically, this study will take place in Badalona and Santa Coloma de Gramenet for the reasons explained below: these are two neighboring municipalities, respectively the second and fourth most populated in Catalonia by the Pakistani population [10], and their Pakistani residents have a similar sociodemographic and socioeconomic profile.

Pakistani women living in these municipalities have the support of two institutions that share the same goal: the integration of migrant people into the host society and its environment. In Badalona, since the 60s, the Ateneu Sant Roc Foundation has worked with the mission of promoting social cohesion in its neighborhood, developing different sociocultural and educational projects for children, young people, and adults, but above all the most vulnerable groups who are at risk of social exclusion, including immigrant women of Pakistani origin. Currently, the Foundation serves approximately 70 Pakistani women of different ages and provides them with personalized support, so that the young Pakistani women receive support in their academic studies and the adult women learn Spanish, Catalan and IT. A large number of Pakistani women are also linked to the Ateneu Sant Roc Foundation's community development projects, in which various socio-cultural and educational promotion activities and socio-health training are carried out.

In Santa Coloma de Gramenet, Casa Asia, which is a cultural center located in Barcelona, started its project "*Aprenem. Families en Xarxa (AFEX)* – (Learning. Families in red)" in different educational centers to promote the integration of people with diverse cultural contexts through the learning of Spanish, Catalan, and IT, with the help of their sons and daughters. Currently, more than 50 Pakistani women living in Santa Coloma de Gramenet are part of the AFEX project. Both institutions have agreed to collaborate in this study, allowing all Pakistani women who have relocated to Barcelona and are affiliated with any of them, to participate in the project.

3.2 Design and Study Population

In this community-based randomized controlled experimental study, any Pakistani woman linked to the collaborating institutions can participate if she meets the established inclusion criteria: adult age (>18 years old), residing in Badalona or Santa Coloma de Gramenet, and voluntarily accepting to participate in the study. Exclusion criteria include living with a participant, having a diagnosis of cognitive impairment or any physical illness that could prevent participation in the study, or disagreeing with the ethical conditions of the study. Considering the number of women served by the collaborating institutions, it was decided to form a sample of 120 women (60 in each entity).

The study will follow a mixed methodological design, combining qualitative and quantitative models and techniques for obtaining and analyzing data.

3.3 The intervention

The development of the study is planned in different phases:

- **Phase 0: Dissemination and Recruitment**

This phase corresponds to the dissemination and recruitment of institutions and participants, which will be started after obtaining the approval of the study by the Bioethics Commission. A detailed project description and request for collaboration will

be included in a cover letter that will be issued to both entities selected to take part in the study. They will also sign an informed consent form. After that, we will be able to begin the recruiting procedure for participants and introduce them to the project. This will involve meetings with the educators and volunteers of these organizations, as well as their Pakistani students, in order to explain the objectives, limitations, and ethical guidelines of the research. During the meetings with the Pakistani women, the communication will be realized in Urdu and Punjabi. After this, a follow-up meeting will be arranged with the interested people to specify details of the project and the implications of participation. Informed consent will be provided to all individuals who wish to participate. The educators of the groups of the collaborating institutions will be in charge of collecting them signed.

Hereafter, both the team and the Pakistani students of the collaborating entities will provide support in the process of dissemination and recruitment, inviting their relatives or acquaintances to participate in the project, until forming the required sample.

- **Phase 1: Data collection**

In this exploratory phase of the project, data will be collected from the participants of both the control and the intervention groups. This data collection will be conducted before starting the intervention (pre-intervention) using a combination of quantitative and qualitative methods and techniques.

From a quantitative perspective, a survey will be administered to determine socio-demographic, clinical, anthropometric, and dietary data. Based on the socio-demographic data (age, education, time of residence in Catalonia, socio-economic factors,...) a representative sample of the participants in each entity will be constructed. From a qualitative perspective and based on the phenomenological method [34], semi-structured interviews will be conducted with some participating women aiming to determine the cultural and religious beliefs related to food along with the strengths and limitations of their current dietary pattern. The information obtained through these interviews will serve, in part, to adjust the educational proposal to the needs and demands of the participants.

Data collection will be conducted on an individual level and, if the pandemic situation permits, in person in a comfortable setting in each entity's room.

- **Phase 2: Implementation of the educational intervention**

In this phase, the food education intervention based on the Transtheoretical model will be implemented, so that in each collaborating entity two main groups of women will be formed (control and intervention) which will later be divided into four subgroups of fifteen women.

The subgroups from the intervention group will receive 10 training sessions over 3 months, while the subgroups of the control group will attend 3 sessions. Considering the cultural and linguistic issues, the sessions will be held in Urdu and Punjabi. The educational materials will also be translated into Urdu. During these sessions, healthy eating habits will be encouraged, bearing in mind the traditional dietary pattern of the study population. The knowledge related to food will be strengthened, taking into account the food beliefs of participants, and their skills will be enhanced to implement healthy eating practices. The food education intervention based on the Transtheoretical model consists of the following:

Table 2. Food education intervention based on the Transtheoretical model

Stage of change	Objectives	Process of change	Change strategy
**Pre-contemplation**	Raise awareness of the problem by stimulating the possibility of change	*Consciousness raising*	Present and discuss food and lifestyle changes due to migration.
		Dramatic relief	Present data on the prevalence of metabolic syndrome and cardiovascular diseases in immigrants of South Asian origin.
		Self-reevaluation	Focus group on the strengths and weaknesses of the dietary pattern.
			Debate on myths and beliefs related to food and health.
		Environmental reevaluation	Highlight the impact of their eating habits on future generations
**Contemplation**	Decant the scale towards change	*Consciousness raising*	Present the benefits of following healthy eating.
			Introduce the disadvantages of following an unhealthy diet.
		Self-reevaluation	Self-assessment of current eating habits by compiling a list of healthy and unhealthy habits.
**Preparation**	Reinforce knowledge to facilitate change	*Counterconditioning*	Conduct group sessions on food or behaviors that need to be enhanced, reduced, or changed regarding the type or quality, to follow a healthy diet.
		Self-liberation	Collect suggestions to acquire the recommended changes.
**Action**	Effectuate the change by promoting self-efficiency	*Stimulus or environmental control*	Workshops on dietary planning and food purchasing; elaboration of healthy dishes with traditional foods; interpretation of nutrition labeling, preparation of healthy breakfasts and snacks.
**Maintenance**	Maintain the change	*Helping relationships*	Photovoice exhibition of healthy plates.
		Social liberation	Acquisition of the role of health promotion agent for the rest of the Pakistani community.

- **Phase 3: Evaluation**

At the completion of the food education sessions, the baseline questionnaire will be readministered to determine whether there has been any change in the variables that were controlled at the start of the study.

After 3 months (short term) the evaluation process will be repeated, in which the women will complete the survey and register their weekly food intake. Anthropometric measurements will also be taken. After 6 months, the mid-term evaluation will take place repeating the same process as the short-term evaluation.

3.4 Variables and Measurement Methods

The variables and methods to be measured will be the following:

- **Age**
  Age will be asked to identify the differences and similarities between different variables.

- **Place of birth**
  Participants will be asked about their birthplace and whether they were born in an urban or rural location.

- **Civil status**
  The options to determine marital status will be the following: single, married, divorced, or widowed.

- **Academic studies**
  The categories to determine the level of study will be the following: illiteracy, early childhood education or equivalent, primary education or equivalent, secondary education or equivalent, vocational training or intermediate level, high school or higher level, and higher education (degree, master's degree and/or doctorate). The country where they studied will also be asked.

- **Occupational status**
  The categories to determine occupational status will be as follows: employed, unemployed, or jobless. Occupations will be identified in both the country of origin and the host country. The profession of the participating women will also be determined.

- **Languages**
  It will be asked which languages both the participating women and their relatives speak.

- **Religion**
  The religion of the participating women will be identified.

- **Reason for migration**
  In the survey, 4 options will be given (family reunification, studies, work reasons, and others) to determine the reason for migration.

- **Years of living in Catalonia**
  The women will state how many years they have lived in Catalonia.

- **Family unit**
  Information regarding the members who are part of the family unit, and their occupations will be requested

- **Socioeconomic factors**
  The monthly income at the family level along with the occupations of different family members will help determine the socioeconomic factors of the sample.

- **Health status**
  To determine the participants' current state of health, they will be asked about past and present illnesses. Particular questions will be asked about some prevalent diseases affecting the Pakistani population, including cardiovascular diseases and the various components of metabolic syndrome. They will be asked to what extent they need to take any medication in order to continue with their daily routine. Finally, they will also be asked if the migration factor has impacted their health and how they define their current health.

- **Nutritional status**
  Nutritional status will be determined through anthropometric measurements. Their body mass index (BMI) will be calculated using their height (cm) and weight (kg). The

classification of the Sociedad Española de Obesidad (SEEDO) will be used for BMI values to define the nutritional status of the participants (Table 3). The normal waist circumference value for South Asian women is ≥ 80 cm.

Table 3. BMI Values

< 18.5	Underweight
**18.5 – 24.9**	Normal weight
**25 – 26.9**	Overweight Grade I
**27 – 29.9**	Overweight Grade II (pre-obesity)
**30 – 34.9**	Obesity Grade 1
**35 – 39.9**	Obesity Grade 2
**40 – 49.9**	Obesity Grade 3 (morbid)

Source: Own elaboration based on SEEDO data.

- **Role in food matters**
  The role in food matters will be studied by identifying the responsibility of cooking, food planning, purchasing, and deciding the menu. It will be asked if women perceive that cooking for the family is their responsibility. On a scale of 10 (with 0 being the least important and 10 being the most important), the factors that influence when deciding on the menu will be studied (cost, season, family preferences, etc.).

- **Dietary knowledge**
  Dietary knowledge will be determined from 14 statements related to some aspects of food with three possible answers: true, false and I don't know.

- **Dietary skills**
  Dietary skills will be studied based on 13 questions about the difficulties that participants have faced in carrying out different actions in aspects of food (planning, buying, preparing food, etc.) during the last 15 days. Answers will be collected with 5 options: 1=No difficulty, 2= Little difficulty, 3= Some difficulty, 4= Strong difficulty, 5= Very difficult.

- **Dietary pattern**
  The dietary pattern will be studied through the survey, in which eating habits will be determined by specifying the time, place, and number of daily meals. Participants will also be asked to answer a food consumption frequency questionnaire inspired by the Guideline Frequencies Table of the Spanish Society of Community Nutrition (SENC) [35]. To complement and deepen the obtained information, the participants will fill in a weekly food record in which they will specify the number, place, and time of the meals, also specifying the type and quantity of the food eaten over the course of a week.

- **Strengths and weaknesses of the dietary pattern**
  The interviews will reveal the differences between the traditional and current dietary patterns. There will also be a description of the factors that make maintaining a healthy diet easier or harder.

- **Food beliefs**
  Through the interview, the beliefs related to the consumption of different foods, their effects on health and well-being, the opinion on naturist products, the myths related to food and health, etc. will be determined.

- **Food improvements**
  Food improvements will be studied through a questionnaire that will be performed during the evaluation phase, in which for each food group worked on during the sessions a question will be asked with five possible answers: (1) Pre-contemplation: "I have not changed my behavior during the last 6 months and neither have planned to do so during the next 6 months." (2) Contemplation: “I have not changed my behavior in the past 6 months, but I am thinking about doing so in the next 6 months.” (3) Preparation: “I am currently trying to change my behavior, but not on a regular basis.” (4) Action: “During the last 6 months, I have already changed my behavior.” (5) Maintenance: "I changed my behavior 6 months ago."

- **Cultural and linguistic adaptation**
  At the end of the sessions, the participants will answer a satisfaction questionnaire translated into Urdu about linguistic, cultural, and content comprehension issues.

4. Ethical aspects

In this study, the established ethical aspects such as the seventh general principle of the Declaration of Helsinki of the World Medical Association (WMA) will be respected. Therefore, an introductory visit before the project commences will be made to the Pakistani users of both institutions. Only the women who sign the informed consent will be able to participate in the project.

During the process of collecting, analyzing, and publishing the data, the anonymity of the participating women will be strictly maintained. All the obtained information will be highly confidential. The intervention will begin after obtaining approval from the Ethics Committee.

5. Applicability of the project

Since Pakistani women are referents to their families in terms of health and nutrition, by receiving culturally and linguistically adapted food education, they will be able to extend it to the rest of the family which may impact positively on the eating habits of future generations. In addition, becoming health-promoting agents will allow them to contribute to strengthening the nutritional and health aspects of their entire community, making the intervention sustainable. The fact that women take the initiative to encourage good eating habits at the family and community levels will provide them with a social and familial role that will help their well-being, which is frequently impacted by the stress of acculturation that these women face.

The nutrition education sessions will be carried out in small groups, so learning will be cooperative, and the group's support will facilitate the change in dietary patterns. In addition, this practice will encourage socialization among Pakistani women who, due to cultural and language barriers, often feel isolated in the host society.

This study will highlight the most common health issues of this ethnic group that have not been explored in Catalonia. A successful implementation of the program, apart from opening future lines of research in the field of health and nutrition for the Pakistani population, can also serve as a model for creating interventions to promote healthy eating habits culturally and linguistically adapted by other minority groups.

6. References

1. Monteiro CA, Cannon G, Levy RB et al. NOVA. The star shines bright. [Food classification. Public health] *World Nutrition* January-March 2016, 7, 1-3, 28-38.
2. Mather, H. M. & Keen, H. (1985) 'The Southhall Diabetes Survey: prevalence of known diabetes in Asians and Europeans', *British Medical Journal* , vol. 291, pp. 1081/1084.
3. McKeigue, P. M., Miller, G. J. & Marmot, M. G. (1989) 'Coronary heart disease in South Asians overseas: a review', *Journal of Clinical Epidemiology*, vol. 42, pp. 597/609.
4. Tillin T, Forouhi N, Johnston DG, McKeigue PM, Chaturvedi N, Godsland IF. Metabolic syndrome and coronary heart disease in South Asians, African-Caribbeans and white Europeans: a UK populationbased cross-sectional study. Diabetologia 2005;48:649-56.
5. Singh, R. B., Niaz, M. A., Ghosh, S., Beegom, R., Agarwal, P., Nangia, S., Moshiri, M. & Janus, E. D. (1998) 'Low fat intake and coronary artery disease in a population with higher prevalence of coronary artery disease: The Indian Paradox', Journal of the American College of Nutrition , vol. 17, pp. 342/350.
6. Farooqi A, Nagra D, Edgar T, Khunti K. Attitudes to lifestyle risk factors for coronary heart disease amongst South Asians in Leicester: a focus group study. *Fam Pract*. 2000 Aug;17(4):293-7.
7. United Nations, Department of Economic and Social Affairs, Population Division (2019). World Population Prospects 2019, Edició online. Rev. 1.
8. Ministeri de pakistanesos d'ultramar i desenvolupament de recursos humans. (2017-18). Year Book. Recuperat de http://www.ophrd.gov.pk/
9. Beltrán J, Sáiz A. La comunidad pakistaní en España. Anu Asia Pacífico CIDOB. 2007;407–16.
10. Institut Nacional d'Estadística. (2020). Población extranjera por país de nacionalidad, edad (grupos quinquenales) y sexo. Recuperat de https://www.ine.es/jaxiT3/Datos.htm?t=36825#!tabs-tabla
11. Institut d'estadística de Catalunya. (2020). Població estrangera per països. Recuperat de https://www.idescat.cat/poblacioestrangera/?b=12
12. Institut d'estadística de Catalunya. (2020). Població estrangera a 1 de gener. Per sexe i edat quinquennal. Recuperat de https://www.idescat.cat/poblacioestrangera/?geo=cat&nac=d426&b=1
13. Hierro-Olavarria, M. A. (2018). *Inmigración y acceso a los servicios públicos: la perspectiva del usuario: Estudio de la problemática comunicativa en el acceso a los*

servicios públicos de las mujeres pakistaníes de Barcelona.Tesi doctoral. Universitat Autònoma de Barcelona, Catalunya.

14. Mellin-Olsen T, Wandel M. Changes in food habits among Pakistani immigrant women in Oslo, Norway. Ethnicity and Health 2005;10:311-39.
15. Khan SA, Jackson RT. The prevalence of metabolic syndrome among low-income South Asian Americans. *Public Health Nutr*. 2016;19:418–28.
16. Dodani S, Henkhaus R, Wick J, Vacek J, Gupta K, Dong L, Butler MG. Metabolic syndrome in South Asian immigrants: more than low HDL requiring aggressive management. *Lipids Health* Dis. 2011;10:45.
17. Kanaya AM, Herrington D, Vittinghoff E, Ewing SK, Liu K, Blaha MJ, Dave SS, Qureshi F, Kandula NR. Understanding the high prevalence of diabetes in U.S. south Asians compared with four racial/ethnic groups: the MASALA and MESA studies. *Diabetes Care*. 2014;37:1621–8.
18. Aryal, N., & Wasti, S. P. (2016). The prevalence of metabolic syndrome in South Asia: a systematic review. *International Journal of Diabetes in Developing Countries*, 36(3), 255-262. https://doi.org/10.1007/s13410-015-0365-5
19. U.P. Gujral, E. Vittinghoff, M. Mongraw-Chaffin, D. Vaidya, N.R. Kandula, M. Allison, J. Carr, K. Liu, K.M.V. Narayan, A.M. Kanaya, Cardiometabolic abnormalities among Normal-weight persons from five racial/ethnic groups in the United States: a cross-sectional analysis of two cohort studies, *Annals of Internal Medicine*. 166 (9) (2017) 628–636.
20. Gulati S, Misra A (2017) Abdominal obesity and type 2 diabetes in Asian Indians: dietary strategies including edible oils, cooking practices and sugar intake. European Journal of Clinical Nutrition 71(7):850–857. https://doi. org/10.1038/ejcn.2017.92
21. Krishnaveni GV, Yajnik CS (2017) Developmental origins of diabetes-an Indian perspective. European Journal of Clinical Nutrition .71(7):865–869. https://doi.org/10.1038/ejcn.2017.87
22. Mellin-Olsen T, Wandel M. Changes in food habits among Pakistani immigrant women in Oslo, Norway. Ethnicity and Health 2005;10:311-39.
23. Kousar, R., Burns, C., & Lewandowski, P. (2008). A culturally appropriate diet and lifestyle intervention can successfully treat the components of metabolic syndrome in female Pakistani immigrants residing in Melbourne, Australia. *Metabolism: Clinical and Experimental*, 57(11), 1502–1508.
24. Choudhry, U. K., Jandu, S., Mahal, J., Singh, R., Sohi-Pabla, H., & Mutta, B. (2002). Health Promotion and Participatory Action Research with South Asian Women. *Journal of Nursing Scholarship, 34(1), 75–81*. doi:10.1111/j.1547-5069.2002.00075.x

25. Bhopal RS, Douglas A, Wallia S, Forbes JF, Lean ME, Gill JM, McKnight JA, Sattar N, Sheikh A, Wild SH, Tuomilehto J, Sharma A, Bhopal R, Smith JB, Butcher I, Murray GD. Effect of a lifestyle intervention on weight change in south Asian individuals in the UK at high risk of type 2 diabetes: a familycluster randomised controlled trial. *Lancet Diabetes Endocrinol*. 2014;2:218–27.
26. Kandula, N.R., Dave, S., De Chavez, P.J. et al. Translating a heart disease lifestyle intervention into the community: the South Asian Heart Lifestyle Intervention (SAHELI) study; a randomized control trial. *BMC Public Health* 15, 1064 (2015). https://doi.org/10.1186/s12889-015-2401-2
27. Jenum et al., (2019). Effects of dietary and physical activity interventions on the risk of type 2 diabetes in South Asians: meta-analysis of individual participant data from randomised controlled trials. *Diabetologia*. 62(8):1337-1348. doi: 10.1007/s00125-019-4905-2.
28. Johansen, K. S., Bjørge, B., Hjellset, V. T., Holmboe-, G., Wandel, M., & Ra, M. (2009). Changes in food habits and motivation for healthy eating among Pakistani women living in Norway : results from the InnvaDiab-DEPLAN study, 13(6), 858–867. https://doi.org/10.1017/S1368980009992047
29. Jafar, T. H., Levey, A. S., White, F. M., Gul, A., Jessani, S., Khan, A. Q., Chaturvedi, N. (2004). Ethnic differences and determinants of diabetes and central obesity among South Asians of Pakistan. Diabetic Medicine, 21(7), 716–723. doi:10.1111/j.1464-5491.2004.01140.x
30. Gask L, Aseem S, Waquas A, Waheed W. Isolation, feeling "stuck" and loss of control: Understanding persistence of depression in British Pakistani women. *Journal of affective Disorders*. 2011;128(1–2).
31. Prochaska, J.O.; Velicer, W.F. The transtheoretical model of health behavior change. Am. J. Heal. Promot. 1997, 12, 38–48, doi:10.4278/0890-1171-12.1.38.
32. Hjellset VT, Ihlebæk CM, Bjørge B, Eriksen HR, Høstmark AT. Health-Related Quality of Life, Subjective Health Complaints, Psychological Distress and Coping in Pakistani Immigrant Women With and Without the Metabolic Syndrome : The InnvaDiab-DEPLAN Study on Pakistani Immigrant Women Living in Oslo, Norway. J Immigr Minor Health. 2011 Aug;13(4):732-41. doi: 10.1007/s10903-010-9409-6.
33. Güell B, Martínez R, Naz K, Solé A. (2018). *Barcelonines d'origen pakistanès: empoderament i participació contra la feminització de la pobresa*. Ajuntament de Barcelona, Catalunya.

34. Høffding, S., Martiny, K. Framing a phenomenological interview: what, why and how. *Phenomenology and the Cognitive Sciences* 15, 539–564 (2016). https://doi.org/10.1007/s11097-015-9433-z
35. Sociedad Española de Nutrición Comunitaria. (2004). Guía de la alimentación saludable. Disponible a Sociedad Española De Nutrición Comunitaria (nutricioncomunitaria.org)
