# Supplementary material for: Effect of the PakCat program on nutrition status, dietary pattern and nutrition knowledge and skills of Pakistani women living in Catalonia evaluated by a mixed-method randomized control trial (RCT)
Source: PLoS One. 2025 Jan 14;20(1):e0316803. doi: 10.1371/journal.pone.0316803 (PMC11731702; doi:10.1371/journal.pone.0316803)
Supplement: S3 File — (PDF) [file pone.0316803.s004.pdf]

DIETARY KNOWLEDGE

Questions	True	False	I don't know
1. To maintain a healthy weight, it is recommended to eliminate all type of fats from the diet.			
2. Eating more than 3 eggs a week increases cholesterol.			
3. Nuts are considered healthy because they contain no fat.			
4. Pulses are a good source of protein.			
5. In a healthy diet, it is advisable to prioritize the consumption of red meat compared to lean meat.			
6. Wholegrain foods are characterized by being rich in fiber.			
7. It is recommended to prioritize the use of whole wheat flour instead of refined flour.			
8. The health benefits of fruit and vegetables are found only in the presence of vitamins and minerals in them.			
9. It is recommended to consume 2 servings of vegetables* and 2 servings of fruit** throughout the day.			
10. Drinking a natural fruit juice is equivalent to eating a piece of fruit.			
11. It is recommended to have more than 2 meals a day.			
12. Following a balanced diet involves eating all foods in the same amounts and proportions.			
13. It is important to drink water only during hot seasons because we sweat more.			
14. Drinking water between or after meals makes us gain weight.			
15. The label of a food product tells us the amount of sugar it contains, among other information.			

* A portion of vegetables is equivalent to 1 plate of cooked vegetables or 1 plate of varied salad or 1 large tomato or 2 carrots or 1 bowl of gazpacho.
** A portion of fruit is equivalent to 1 large piece of fruit or 2 small pieces or 1 cup of small fruit or 1 slice of pineapple, melon, watermelon...

SKILLS IN FOOD-RELATED ASPECTS

The following questions refer to the difficulty you have experienced in doing each of these tasks during the last 15 days. Indicate a value from 1 to 5 depending on the degree of difficulty.

☐No difficulty ☐Little difficulty ☐Some difficulty ☐Quite a bit of difficulty ☐A lot of difficulties

- Planning menus in advance.

No difficulty ☐1 ☐2 ☐3 ☐4 ☐5 A lot of difficulty

- Making the shopping list.

No difficulty ☐1 ☐2 ☐3 ☐4 ☐5 A lot of difficulty

- Choosing between the different varieties of foods within the same typology.

No difficulty ☐1 ☐2 ☐3 ☐4 ☐5 A lot of difficulty

- Choosing foods considering their season.

No difficulty ☐1 ☐2 ☐3 ☐4 ☐5 A lot of difficulty

- Understanding the nutritional information on product labels to make a healthy decision when purchasing food.

No difficulty ☐1 ☐2 ☐3 ☐4 ☐5 A lot of difficulty

- Preparing and cooking dishes using known ingredients.

No difficulty ☐1 ☐2 ☐3 ☐4 ☐5 A lot of difficulty

- Cooking by following a recipe.

No difficulty ☐1 ☐2 ☐3 ☐4 ☐5 A lot of difficulty

- Modifying a recipe if a specific ingredient is not available.

No difficulty ☐1 ☐2 ☐3 ☐4 ☐5 A lot of difficulty

- Using different cooking methods (grilling, boiling, baking, sautéing, etc.) throughout the week.

No difficulty ☐1 ☐2 ☐3 ☐4 ☐5 A lot of difficulty

- Reusing leftover food to prepare new meals.

No difficulty ☐1 ☐2 ☐3 ☐4 ☐5 A lot of difficulty

- Preparing a balanced meal with different types of foods.

No difficulty ☐1 ☐2 ☐3 ☐4 ☐5 A lot of difficulty

DIETARY PATTERN

How often do you consume the following foods? (Mark only one option)

	Rarely/Never	Less than once a month	1-3 times a month	1-2 times a week	3-4 times a week	5-6 times a week	1-2 times a day	≥ 3 times a day
Whole grains (whole wheat flour, brown rice, oats, ...)								
Refined grains (refined wheat flour, pasta, rice, ...)								
Fruit								
Raw or cooked vegetables								
Dairy products (yogurt, milk, cheese, ...)								
Nuts								
Pulses (chickpeas, lentils, beans, ...)								
Eggs								
Fish or seafood								
White meat (chicken, turkey, rabbit, ...)								
Red meat (beef, lamb, ...)								
Processed meat (sausages, ...)								
Carbonated drinks (Fanta, Coca-Cola, ...)								
Packaged fruit juices								
Fast food (pizza, hamburgers, ...)								
Pre-cooked foods (croquettes, pasties, ...)								
Salty snacks (potato chips, ...)								
Industrial or homemade pastries (cookies, cakes, ...)								

CONEIXEMENTS DIETÈTICS

Preguntes	Cert	Fals	No ho sé
1. Per a mantenir un pes saludable s'aconsella eliminar tots els greixos de la dieta.			
2. Menjar més de 3 ous a la setmana incrementa el colesterol.			
3. Els fruits secs es consideren saludables perquè no contenen greix.			
4. Els llegums són una bona font de proteïna.			
5. En una alimentació saludable, s'aconsella prioritzar el consum de carn vermella en comparació amb la carn magra.			
6. Els aliments integrals es caracteritzen per ser rics en fibra.			
7. S'aconsella prioritzar l'ús de la farina integral en lloc de la farina refinada.			
8. Els beneficis per a la salut de la fruita i la verdura es troben únicament en la presència de les vitamines i minerals.			
9. Es recomana consumir 2 racions de verdura* i 2 de fruita** al llarg del dia.			
10. Prendre un suc de fruita natural és equivalent a menjar una peça de fruita.			
11. S'aconsella fer més de 2 àpats al dia.			
12. Seguir una dieta equilibrada implica menjar tots els aliments en les mateixes quantitats i proporcions.			
13. És important beure aigua únicament en les èpoques que fa més calor perquè es sua més.			
14. Beure aigua entre o després dels àpats engreixa.			
15. L'etiqueta d'un producte alimentari ens indica la quantitat de sucre que conté un aliment, entre d'altre informació.			

* Una ració de verdura equival a 1 plat de verdura cuita o bé 1 plat d'amanida variada o bé 1 tomàquet gran o bé 2 pastanagues o bé 1 bol de gaspatxo.
** Una ració de fruita equival a 1 peça gran de fruita o bé 2 peces petites o bé 1 tassa de fruita menuda o bé 1 tall de pinya, meló, síndria...

HABILITATS EN ASPECTES RELACIONATS AMB L'ALIMENTACIÓ

Les preguntes que segueixen a continuació es refereixen a la dificultat que ha tingut per a realitzar cada una de les diferents accions en els últims 15 dies. Indiqui un valor del 1 al 5 en funció el grau de dificultat.

☐*Cap dificultat* ☐ *Poca dificultat* ☐*Alguna dificultat* ☐*Força dificultat* ☐*Molta dificultat*

- Planificar els menús amb antelació.
  Cap dificultat ☐ ☐1 ☐2 ☐3 ☐4 5 Molta dificultat
- Elaborar la llista de la compra.
  Cap dificultat ☐ ☐1 ☐2 ☐3 ☐4 5 Molta dificultat
- Escollir entre les diferents varietat d'aliments dins d'una mateixa tipologia.
  Cap dificultat ☐ ☐1 ☐2 ☐3 ☐4 5 *Molta dificultat*
- Escollir els aliments tenint en compte la seva temporada.
  Cap dificultat ☐ ☐1 ☐2 ☐3 ☐4 5 *Molta dificultat*
- Comprendre la informació nutricional de les etiquetes dels productes alimentaris per prendre una decisió saludable a l'hora de comprar.
  Cap dificultat ☐ ☐1 ☐2 ☐3 ☐4 5 *Molta dificultat*
- Preparar i cuinar plats a partir d'ingredients coneguts.
  Cap dificultat ☐ ☐1 ☐2 ☐3 ☐4 5 *Molta dificultat*

- Cuinar seguint una recepta.

Cap dificultat ☐ ☐1 ☐2 ☐3 ☐4 5 *Molta dificultat*

- Modificar una recepta si no es té algun ingredient concret.

Cap dificultat ☐ ☐1 ☐2 ☐3 ☐4 5 *Molta dificultat*

- Utilitzar diferents mètodes de cocció (planxa, bullit, forn, saltejat,...) al llarg de la setmana. ☐ ☐ ☐ ☐ ☐

Cap dificultat 1 2 3 4 5 *Molta dificultat*

- Reutilitzar aliments sobrats per a preparar nous àpats.

Cap dificultat ☐ ☐1 ☐2 ☐3 ☐4 5 *Molta dificultat*

- Preparar un àpat equilibrat amb presència de diferents varietats d'aliments.

Cap dificultat ☐ ☐1 ☐2 ☐3 ☐4 5 *Molta dificultat*

PATRÓ DIETÈTIC

- **Amb quina freqüència consumeix els següents aliments:** (Marqui només una creu)

	Rarament/ Mai	Menys d'1 vegada/mes	1-3 vegades/ mes	1-2 vegades/ setmana	3-4 vegades/ setmana	5-6 vegades/setma na	1-2 vegades/ dia	≥ 3 vegades/ dia
Cereals integrals (farina integral, arròs integral, civada, ...)								
Cereals refinats (farina refinada de blat, pasta, arròs, ...)								
Fruita								
Verdura crua o cuita								
Làctics (Iogurt, llet, formatge, ...)								
Fruits secs								
Llegums (cigrons, llenties, mongetes,..)								
Ous								
Peix o marisc								
Carn blanca (pollastre, gall dindi, conill,..)								
Carn vermella (vedella, xai,...)								
Carn processada (embotits,..)								
Begudes carbonatades (Fanta, Coca-cola,...)								
Sucs de fruita envasats								
Menjar ràpid (pizza, hamburguesa,...)								
Aliments precuinats (croquetes, empanades,..)								
Snacks salats (patates xips,..)								
Pastisseria industrial o casera (galetes, pastissos, ...)								
