# Supplementary material for: Effect of the PakCat program on nutrition status, dietary pattern and nutrition knowledge and skills of Pakistani women living in Catalonia evaluated by a mixed-method randomized control trial (RCT)
Source: PLoS One. 2025 Jan 14;20(1):e0316803. doi: 10.1371/journal.pone.0316803 (PMC11731702; doi:10.1371/journal.pone.0316803)
Supplement: S4 File — (PDF) [file pone.0316803.s005.pdf]

Scoring criteria for the Food Frequency Questionnaire (FFQ)

	Rarely/Never	Less than once a month	1-3 times a month	1-2 times a week	3-4 times a week	5-6 times a week	1-2 times a day	≥ 3 times a day
Whole grains (whole wheat flour, brown rice, oats, ...)	0	0	0	0	0	0	1	1
Refined grains (refined wheat flour, pasta, rice, ...)	1	1	1	0	0	0	0	0
Fruit	0	0	0	0	0	0	0	1
Raw or cooked vegetables	0	0	0	0	0	0	1	1
Dairy products (yogurt, milk, cheese, ...)	0	0	0	0	0	0	1	1
Nuts	0	0	0	0	1	1	1	0
Pulses (chickpeas, lentils, beans, ...)	0	0	0	0	1	1	0	0
Eggs	0	0	0	0	1	1	0	0
Fish or seafood	0	0	0	1	1	0	0	0
White meat (chicken, turkey, rabbit, ...)	0	0	0	1	1	0	0	0
Red meat (beef, lamb, ...)	0	0	0	1	0	0	0	0
Processed meat (sausages, ...)	1	1	1	0	0	0	0	0
Carbonated drinks (Fanta, Coca-Cola, ...)	1	1	1	0	0	0	0	0
Packaged fruit juices	1	1	1	0	0	0	0	0
Fast food (pizza, hamburgers, ...)	1	1	1	0	0	0	0	0
Pre-cooked foods (croquettes, pasties, ...)	1	1	1	0	0	0	0	0
Salty snacks (potato chips, ...)	1	1	1	0	0	0	0	0
Industrial or homemade pastries (cookies, cakes, ...)	1	1	1	0	0	0	0	0

Criteris de puntuació per Qüestionar de Freqüència de Consum Alimentari (QFCA)

	Rarament/ Mai	Menys d'1 vegada/mes	1-3 vegades/ mes	1-2 vegades/ setmana	3-4 vegades/ setmana	5-6 vegades/setmana	1-2 vegades/ dia	≥ 3 vegades/ dia
**Cereals integrals (farina integral, arròs integral, civada, ...)**	0	0	0	0	0	0	1	1
**Cereals refinats (farina refinada de blat, pasta, arròs, ...)**	1	1	1	0	0	0	0	0
**Fruita**	0	0	0	0	0	0	0	1
**Verdura crua o cuita**	0	0	0	0	0	0	1	1
**Làctics (Iogurt, llet, formatge, ...)**	0	0	0	0	0	0	1	1
**Fruits secs**	0	0	0	0	1	1	1	0
**Llegums (cigrons, llenties, mongetes,..)**	0	0	0	0	1	1	0	0
**Ous**	0	0	0	0	1	1	0	0
**Peix o marisc**	0	0	0	1	1	0	0	0
**Carn blanca (pollastre, gall dindi, conill,..)**	0	0	0	1	1	0	0	0
**Carn vermella (vedella, xai,...)**	0	0	0	1	0	0	0	0
**Carn processada (embotits,..)**	1	1	1	0	0	0	0	0
**Begudes carbonatades (Fanta, Coca-cola,...)**	1	1	1	0	0	0	0	0
**Sucs de fruita envasats**	1	1	1	0	0	0	0	0
**Menjar ràpid (pizza, hamburguesa,...)**	1	1	1	0	0	0	0	0
**Aliments precuinats (croquetes, empanades,..)**	1	1	1	0	0	0	0	0
**Snacks salats (patates xips,..)**	1	1	1	0	0	0	0	0
**Pastisseria industrial o casera (galetes, pastissos, ...)**	1	1	1	0	0	0	0	0
